# The transcription factor NHR-8: A new target to increase ivermectin efficacy in nematodes

**DOI:** 10.1371/journal.ppat.1007598

**Published:** 2019-02-13

**Authors:** Cécile Ménez, Mélanie Alberich, Elise Courtot, Fabrice Guegnard, Alexandra Blanchard, Hugo Aguilaniu, Anne Lespine

**Affiliations:** 1 INTHERES, Université de Toulouse, INRA, ENVT, Toulouse, France; 2 INRA, UMR 1282 Infectiology and Public Health, Nouzilly, Université François Rabelais de Tours, France; 3 CNRS, detached to the Serrapilheira Institute, Rio de Janeiro, Brazil; Iowa State University, UNITED STATES

## Abstract

Resistance to the anthelmintic macrocyclic lactone ivermectin (IVM) has a great impact on the control of parasitic nematodes. The mechanisms by which nematodes adapt to IVM remain to be deciphered. We have identified NHR-8, a nuclear hormone receptor involved in the xenobiotic response in *Caenorhabditis elegans*, as a new regulator of tolerance to IVM. Loss-of-function *nhr-8(ok186) C*. *elegans* mutants subjected to larval development assays and electropharyngeogram measurements, displayed hypersensitivity to IVM, and silencing of *nhr-8* in IVM-resistant worms increased IVM efficacy. In addition, compared to wild-type worms, *nhr-8* mutants under IVM selection pressure failed to acquire tolerance to the drug. In addition, IVM-hypersensitive *nhr-8(ok186)* worms displayed low transcript levels of several genes from the xenobiotic detoxification network and a concomitant low Pgp-mediated drug efflux activity. Interestingly, some *pgp* and *cyp* genes known to impact IVM tolerance in many nematode species, were down regulated in *nhr-8* mutants and inversely upregulated in IVM-resistant worms. Moreover, *pgp-6* overexpression in *nhr-8(ok186) C*. *elegans* increased tolerance to IVM. Importantly, NHR-8 function was rescued in *nhr-8(ok186) C*. *elegans* with the homolog of the parasitic nematode *Haemonchus contortus*, and silencing of *Hco-nhr-8* by RNAi on L2 *H*. *contortus* larvae increased IVM susceptibility in both susceptible and resistant *H*. *contortus* isolates. Thus, our data show that NHR-8 controls the tolerance and development of resistance to IVM in *C*. *elegans* and the molecular basis for this relates to the NHR-8-mediated upregulation of IVM detoxification genes. Since our results show that *Hco*-*nhr-8* functions similarly to *Cel*-*nhr-8*, this study helps to better understand mechanisms underlying failure in drug efficacy and open perspectives in finding new compounds with NHR-8 antagonist activity to potentiate IVM efficacy.

## Author summary

IVM is the most important broad-spectrum deworming drug used today but resistance to this drug has appeared in parasites of both animals and humans. This seriously jeopardizes the success of current parasite control. Preserving IVM efficacy is a public health issue, whose outcome depends on the understanding of the molecular basis of selection for resistance to these drugs. We unambiguously show that the nuclear hormone receptor NHR-8, is crucial for protection of the nematode model *Caenorhabditis elegans* against IVM toxicity. Worms deficient in NHR-8 are hypersensitive to IVM and fail to become resistant to IVM under drug pressure. NHR-8 functions in the parasitic nematode of ruminants *Haemonchus contortus* and similar mechanisms could occur in other target pathogens. By controlling the xenobiotic detoxification network, NHR-8 may contribute to the biotransformation and elimination of IVM and help to desensitize the worm to the drug. This provides novel molecular targets involved in IVM drug tolerance in parasitic nematodes. Such findings could be exploited for targeted therapeutic intervention to treat parasitic nematode infections and delay the process of resistance development to anthelmintic drugs.

## Introduction

Helminth infections affect nearly three billion people worldwide and cause the highest economic losses in livestock due to lower productivity. Ivermectin (IVM) is an antiparasitic compound, belonging to the macrocyclic lactone (ML) family, used worldwide for the control of endo- and ecto-parasites in veterinary and human medicine [1]. IVM was first approved for use in the veterinary field and remains the only ML available for mass chemotherapy in human onchocerciasis and strongyloidiasis. Unfortunately, IVM resistance is rampant in parasites of ruminants and horses [2,3], and IVM loss-of-efficacy is now observed in dog [4] and human parasites [5].

Because of the increasing limitations of existing products, there is an urgent need to discover new agents active against resistant parasitic nematodes and to identify additional biological targets. Innovation in anthelmintic discovery will depend on advancing knowledge on the mechanisms involved in the development of drug resistance in nematodes.

There is now considerable evidence that ML resistance in nematodes has a multigenic basis [6,7]. In addition, changes in xenobiotic detoxification performance is among one important mechanism of multidrug resistance in pathogens. The expression or increase in the frequency of alleles correlated with resistance of genes encoding ATP-binding-cassette (ABC) transporters such as P-glycoproteins (PGPs), and phase I and phase II detoxifying enzymes, are modified in IVM-resistant helminths of veterinary importance [8–13]. Accordingly, exposure to MLs in nematodes results in increased transcription of multiple genes involved in xenobiotic metabolism and transport, both *in vitro* [10,12,14–16] and *in vivo* [17–19]. This suggests increased ML detoxification, and consequently decreased drug efficacy that enable parasites to survive ML exposure and may contribute to selection for resistant worms.

In mammals, IVM also induces gene expression and activity of ABC transporters and cytochromes involved in its own metabolism, such as PGP and cytochrome CYP1A, 2B and 3A isoenzymes *in vitro* [20,21] and *in vivo* [21–23], but little is currently known about the molecular mechanisms of these regulations. Generally, expression of genes involved in drug detoxification is under the control of few ligand-activated transcription factors and nuclear receptors referred to as "xenosensors": CAR (Constitutive androstane receptor), PXR (Pregnane X receptor), as well as the farnesoid X receptor (FXR), the liver X receptor (LXR), the aryl-hydrocarbon receptor (AHR) and the vitamin D receptor (VDR) [24]. These transcription factors are activated by various xenobiotics (contaminants, drugs or dietary compounds), leading to their binding to response elements located on the promoters of target genes and to the modulation of the expression of these genes. While IVM does not display PXR, CAR nor AhR ligand activities [20], it was shown to be a partial agonist of FXR [25], and it might thereby regulate the expression of FXR-target genes such as PGPs and cytochromes, that are important in endogenous and exogenous lipid metabolism.

Although the free-living nematode *C*. *elegans* possesses detoxifying systems that are responsive to xenobiotics [26,27], the regulators of the transcriptional response following an IVM exposure have not been yet explored. *C*. *elegans* possess an impressive battery of 284 Nuclear Hormone Receptors (NHRs), of which only 15 correspond to “conserved” NHRs that have clear homologs in other species [28]. Interestingly, DAF-12, NHR-8 and NHR-48 are structurally related to the mammalian xenosensors and appear to be the most relevant for xenobiotic metabolism regulation in *C*. *elegans* [29,30]. NHR-8 and DAF-12 are both clearly involved in steroid homeostasis, growth and lifespan, and are considered as FXR and LXR functional homologs [31–33]. While NHR-8 is known for regulation of xenobiotic metabolism in *C*. *elegans* [30], DAF-12 and NHR-48 have not yet been studied regarding this possible function.

In the general context of determining the molecular factors that govern IVM tolerance (i.e. decreased efficacy), we have evaluated the specific role of DAF-12, NHR-8 and NHR-48 using *C*. *elegans* as a nematode model. We evaluated the susceptibility to IVM of strains lacking these individual nuclear receptors. Since NHR-8 was discovered to specifically determine IVM tolerance, we explored its role in the transcriptional regulation of the drug detoxification network and identified some genes that were specifically regulated by NHR-8, that are also important for IVM tolerance. We then analyzed the involvement of *nhr-8* in the adaptive response of *C*. *elegans* to IVM in the environment. Finally, we evaluated its conserved function in *Haemonchus contortus*, a pathogenic parasitic nematode economically relevant in livestock. This study identifies for the first time the nuclear hormone receptor NHR-8 as a key regulator of IVM tolerance in both the free-living nematode *C*. *elegans* and *H*. *contortus*.

## Results

### *nhr-8* loss-of-function *C*. *elegans* are hypersensitive to IVM

**Fig 1:**
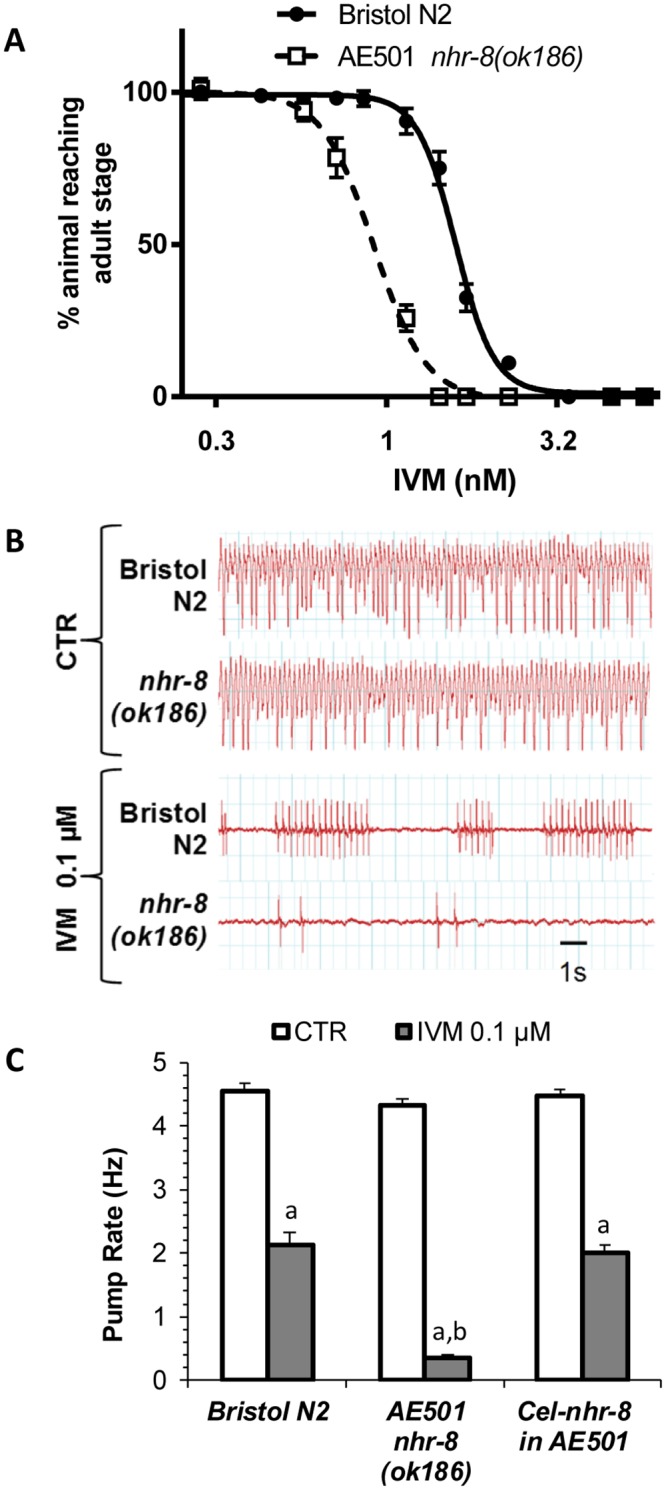
Effects of loss of NHR-8 function on susceptibility of *Caenorhabditis elegans* to IVM. (A) Dose response curves to IVM in a larval development assay of *nhr-8(ok186)* in comparison to the wild-type Bristol N2. Values represent the percentage of L1 reaching the young adult stage after 55 hours of incubation at 21°C within the presence of increasing doses of IVM. Data are mean ± SEM from 6 independent experiments. (B) Microfluidic Electropharyngeograms (EPGs) recorded from wild-type N2 Bristol and *nhr-8(ok186)* with or without IVM exposure. (C) Analysis of pharyngeal pumping activity (pump frequency) showing hypersensitivity of *nhr-8(ok186)* mutant to IVM and the rescue of IVM sensitivity by *C*. *elegans nhr-8* cDNA. Pump frequency was compared in worms exposed to control or 0.1 μM IVM using the Nemametrix ScreenChip system. EPG recordings (2–4 min per worm) were started 20 min after the onset of IVM exposure. Data are reported as the mean ± SEM; n = 30–90 worms/group. a p<0.001 *vs* untreated worms; b p<0.001 *vs* wild-type Bristol N2.

In order to identify transcriptional regulators that could be involved in susceptibility to IVM in *C*. *elegans*, three strains (AE501 *nhr-8(ok186)*, AA107 *nhr-48(ok178)* and DR20 *daf-12(m20)*), each lacking function of individual NHRs, NHR-8, NHR-48 and DAF-12 respectively, were subjected to a larval development assay in the presence of the drug. EC_50_s, i.e. the concentrations of compound at which 50% of the animals fail to reach the adult stage, are shown in Table 1. Among these three strains, only the strain displaying *nhr-8* loss-of-function showed increased IVM susceptibility compared with the wild-type Bristol N2 strain. This is revealed by a 2-fold lower EC_50_ value (0.96 ± 0.12 vs 1.63 ± 0.29 nM, p<0.001, Table 1) and a significant shift to the left of the dose-response curves (Fig 1A).

**Table 1:** Susceptibilities of wild-type Bristol N2 strain and individual NHRs loss-of-function *Caenorhabditis elegans* mutants to IVM.

Strain	EC_50_ IVM (nM)
Bristol N2	1.63 ± 0.29
AE501, *nhr-8(ok186)*	0.96 ± 0.12 ***
AA107, *nhr-48(ok178)*	1.62 ± 0.14
DR20, *daf-12(m20)*	1.67 ± 0.17

EC_50_ (effective concentration for 50% inhibition) calculated from Larval Development Assay.

*** p<0.001 vs Bristol N2

Larval development assays with two other *nhr-8* loss-of-function mutants, *nhr-8(hd117)* and *nhr-8(tm1800*), confirmed the specific role of *nhr-8* on the sensitivity of worms against IVM (S1 Fig). We therefore focused on *nhr-8* and used the *nhr-8(ok186)* mutant in the following experiments.

Since IVM inhibits pharyngeal pumping of nematodes through action on glutamate-gated chloride channels (GluCls) [34], the impact of loss of *nhr-8* on the reduction in pharyngeal pumping was investigated (Fig 1B&1C). A 20 min-exposure with 0.1 μM IVM of the wild-type *C*. *elegans* induced a 2.4-fold decrease in pharyngeal pumping rate while the effect of the same IVM concentration on *nhr-8(ok186)* mutant worms was dramatically stronger with a 13-fold decrease in pharyngeal pumping rate (Fig 1C, p<0.001 vs the wild-type). In order to confirm that this hypersensitive phenotype to IVM could be reversed by expression of the gene mutated in the strain AE501 (*nhr-8(ok186)*), we transformed AE501 with cDNA encoding *Cel-nhr-8* under the control of the *nhr-*8 promoter. Transformation with *Cel-nhr-8* resulted in complete rescue of IVM susceptibility (Fig 1C). Similar results were obtained with two different lines expressing the *C*. *elegans* gene. Altogether, these results clearly indicated a hypersensitivity of *nhr-8*-deficient worms to IVM.

### *nhr-8* silencing increases IVM susceptibility in wild-type and ML-resistant *C*. *elegans* strains

**Fig 2:**
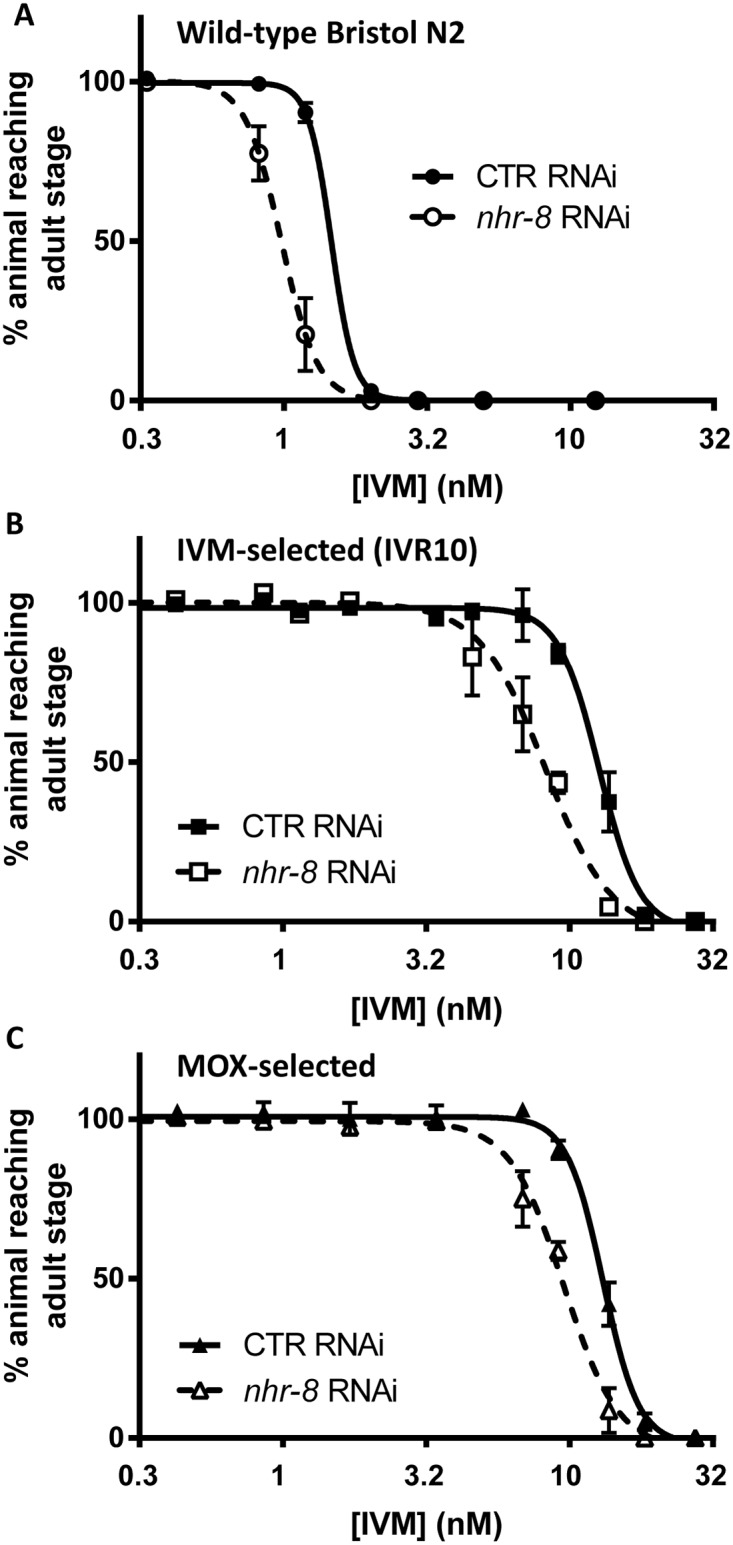
Effects of *nhr-8* silencing on susceptibilities of wild-type N2 Bristol (A), IVM-selected (IVR10) (B) and MOX-selected (C) *C*. *elegans* strains to IVM in a larval development assay. Worms were fed on HT115 bacteria transformed whether with L4440 vector that produces double-stranded RNA against the *Cel-nhr-8* gene, or with the empty vector as control. Values for dose response curves to IVM of IVM- and MOX-selected worms, both IVM-resistant, and wild-type represent the percentage of L1 reaching the young adult stage after 55 hours of incubation at 21°C within the presence of increasing doses of IVM. Data are mean ± SD from 4–6 independent experiments. Efficiencies of *nhr-8* knockdown are presented in S2 Fig and S2 Table.

We then investigated the involvement of *nhr-8* in the response to IVM in wild-type and ML-tolerant *C*. *elegans* strains using gene-specific silencing via RNAi. Two ML-selected strains both resistant to IVM were used. These strains have been generated by stepwise exposure with either IVM or moxidectin (MOX) selection pressure, as previously described [10,15]. Silencing of *nhr-8* induced an increase in IVM efficacy in both wild-type and resistant *C*. *elegans* strains, as revealed by a significant shift to the left (low EC_50_) of the dose-response curves, compared with the control RNAi (Fig 2A, 2B & 2C). EC_50_s of IVM from wild-type, IVM- and MOX-selected strains on *nhr-8* RNAi were reduced compared with control conditions from 1.5 ± 0.3 nM to 1.0 ± 0.2 nM (Table 2, p<0.05), 12.7 ± 1.2 to 7.1 ± 2.1 nM (Table 2, p<0.01) and from 13.1 ± 0.4 nM to 8.8 ± 1.6 nM (Table 2, p<0.05), respectively. As a result, *nhr-8* RNAi animals from IVM-selected and MOX-selected strains were 1.8- and 1.5-fold more sensitive to IVM, respectively (Table 2). Consequently, when *nhr-8* was silenced, the resistance factor was lowered from 8.7 to 4.8 in the IVM-selected strain and from 8.9 to 6.0 in the MOX-selected strain. These results clearly show that *nhr-8* is a key factor involved in IVM tolerance in both susceptible and resistant *C*. *elegans*.

**Table 2:** Effect of *nhr-8* silencing on susceptibilities of wild-type Bristol N2, IVM-selected (IVR10) and MOX-selected *C*. *elegans* strain to IVM.

	Bristol N2	IVM-selected strain (IVR10)		MOX-selected strain	
	EC_50_ IVM (nM)	EC_50_ IVM (nM)	RF	EC_50_ IVM (nM)	RF
Control RNAi	1.5 ± 0.3	12.7 ± 1.2	8.7	13.1 ± 0.4	8.9
*nhr-8* RNAi	1.0 ± 0.2 *	7.1 ± 2.1 **	4.8	8.8 ± 1.6 *	6.0
R	1.3	1.8		1.5	

EC_50_ (effective concentration for 50% inhibition) calculated from LDA.

RF (Resistance factor) is the fold resistance relative to the wild-type Bristol N2 strain.

R is the ratio of the EC50 of IVM with control RNAi to the EC50 of IVM with *nhr-8* RNAi.

** p<0.01;

* p<0.05 vs Control RNAi. Data are mean ± S.D. from 4–6 independent experiments.

### *nhr-8* loss depresses expression of drug detoxification genes in *C*. *elegans*

**Fig 3:**
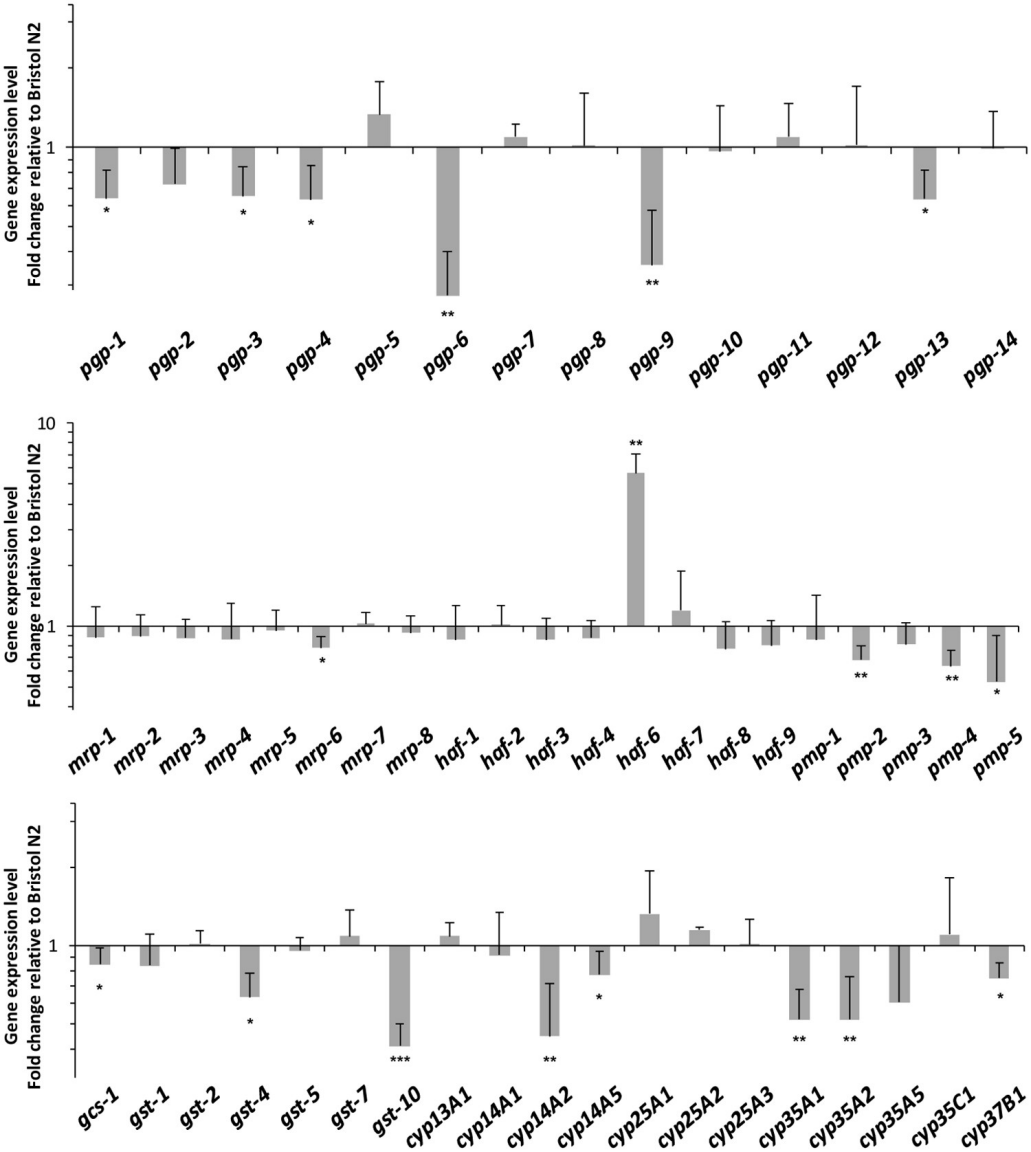
Transcriptional profile of genes associated with xenobiotic metabolism and transport in *nhr-8(ok186) C*. *elegans* relative to wild-type worms. Changes in levels of mRNAs encoding ABC transporters and several phase II detoxification enzymes and cytochromes P450, normalized with respect to *cdc-42* mRNA levels, were determined by real-time qPCR. Gene expression levels are expressed as -fold change relative to wild-type Bristol N2 and are reported as the mean ± S.D. of four to five independent experiments. * p<0.05; ** p<0.01; *** p<0.001 vs. wild-type Bristol N2. Student’s t-test between wild-type and *nhr-8* mutant for each gene.

Since IVM efficacy is strongly related to the final concentration of the drug in the worm, which highly depends on the performance of xenobiotic metabolism systems, we next assessed the impact of a disruption of *nhr-8* on the transcriptional profiles of genes typically involved in xenobiotic metabolism and transport. Fig 3 shows the gene expression level of potential target genes of NHR-8 among xenobiotic detoxification system in *nhr-8* deficient worms relative to the wild-type. Significant decreased expression in 18 genes was observed in *nhr-8(ok186)*, while only one (*haf-6*) showed increased expression compared with wild-type worms, suggesting that they are regulated in a *nhr-8*-dependent manner. Interestingly, several Phase III genes belonging to ABC transporter family were downregulated in *nhr-8* deficient worms, namely *pgp-6* (3.7-fold), *pgp-9* (2.7-fold), *pmp-5* (1.9-fold), *pgp-4* (1.6-fold), *pmp-4* (1.6-fold), *pgp-3* (1.5-fold), *pgp-*13 (1.5-fold), *pmp-2* (1.5-fold), *pgp-1* (1.4-fold), and *mrp-6* (1.3-fold), compared with wild-type worms. Besides, a reduced expression of genes encoding some Phase I and Phase II detoxification enzymes namely *cyp14A2* (2.2-fold), *cyp35A1* (2.0-fold), *cyp35A2* (2.0-fold), *cyp14A5* (1.4-fold), *cyp37B1* (1.4-fold) and *gst-10* (2.4-fold), *gst-4* (1.6-fold), *gcs-1* (1.3-fold) was observed in *nhr-8* mutants compared with wild-type animals. These results support that *nhr-8* has a role in xenobiotic response pathways since many potential detoxifying enzymes may have lower activity in *nhr-8* deficient mutants.

**Fig 4:**
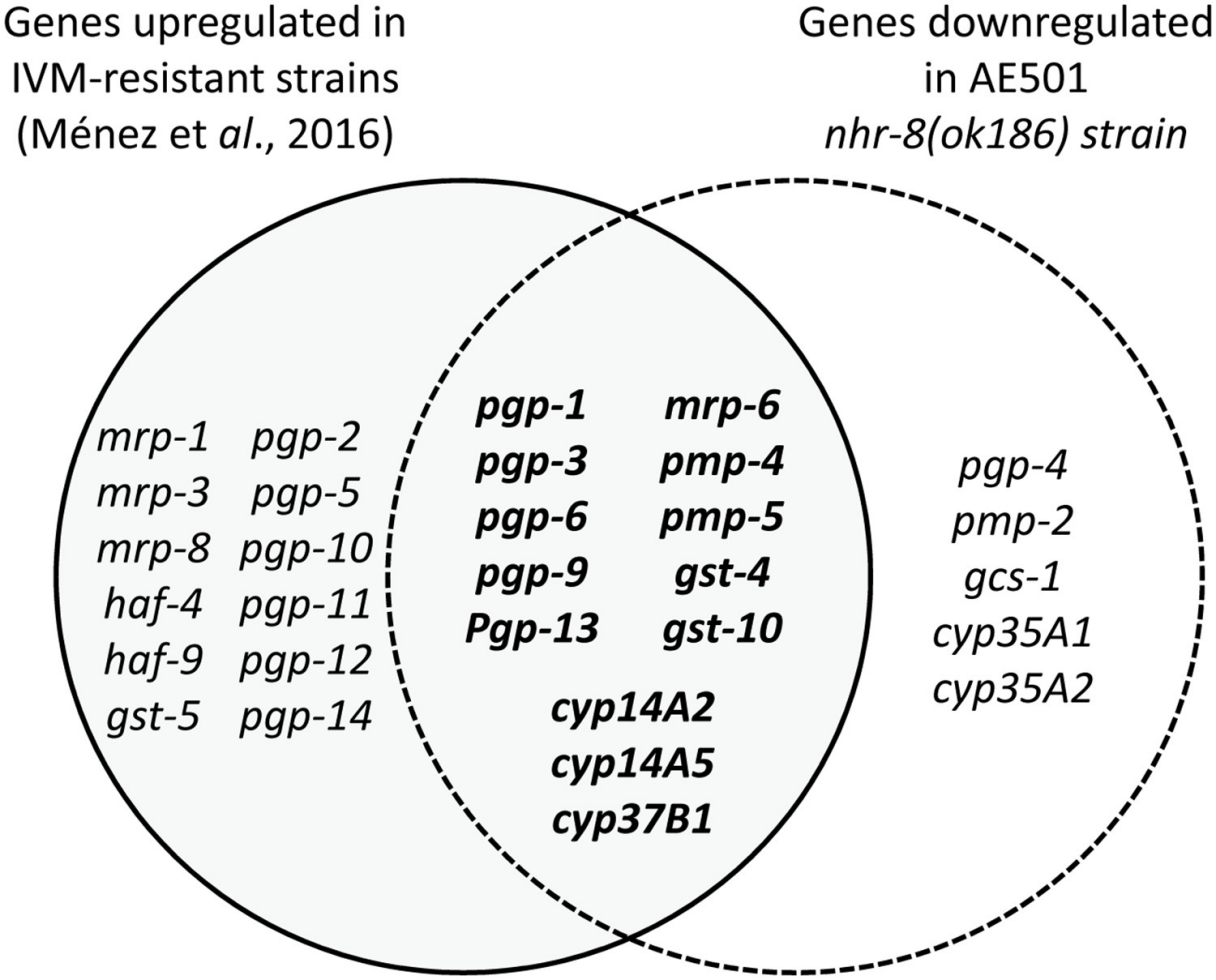
Venn diagram showing the overlap between genes upregulated in IVM-resistant strains and genes downregulated in *nhr-8(ok186)* mutants.

In order to search for relevant *nhr-8* target genes that are crucial for IVM tolerance, we built a Venn diagram comparing constitutive expression level of detoxification genes in IVM-hypersensitive *nhr-8*-deficient worms and in IVM-resistant worms [15]. Interestingly, we identified several relevant candidates from the ABC transporter family (*pgp-1*, *pgp-3*, *pgp-6*, *pgp-9*, *pgp-13*, *mrp-6*, *pmp-4*, *pmp-5)* as well as some GSTs and CYPs *(gst-4*, *gst-10*, *cyp14A2*, *cyp14A5* and *cyp37B1)* that were upregulated in IVM-resistant worms, and inversely downregulated in *nhr-8*-deficient worms (Fig 4).

### *nhr-8* loss increases PGP substrate accumulation in *C*. *elegans*

**Fig 5:**
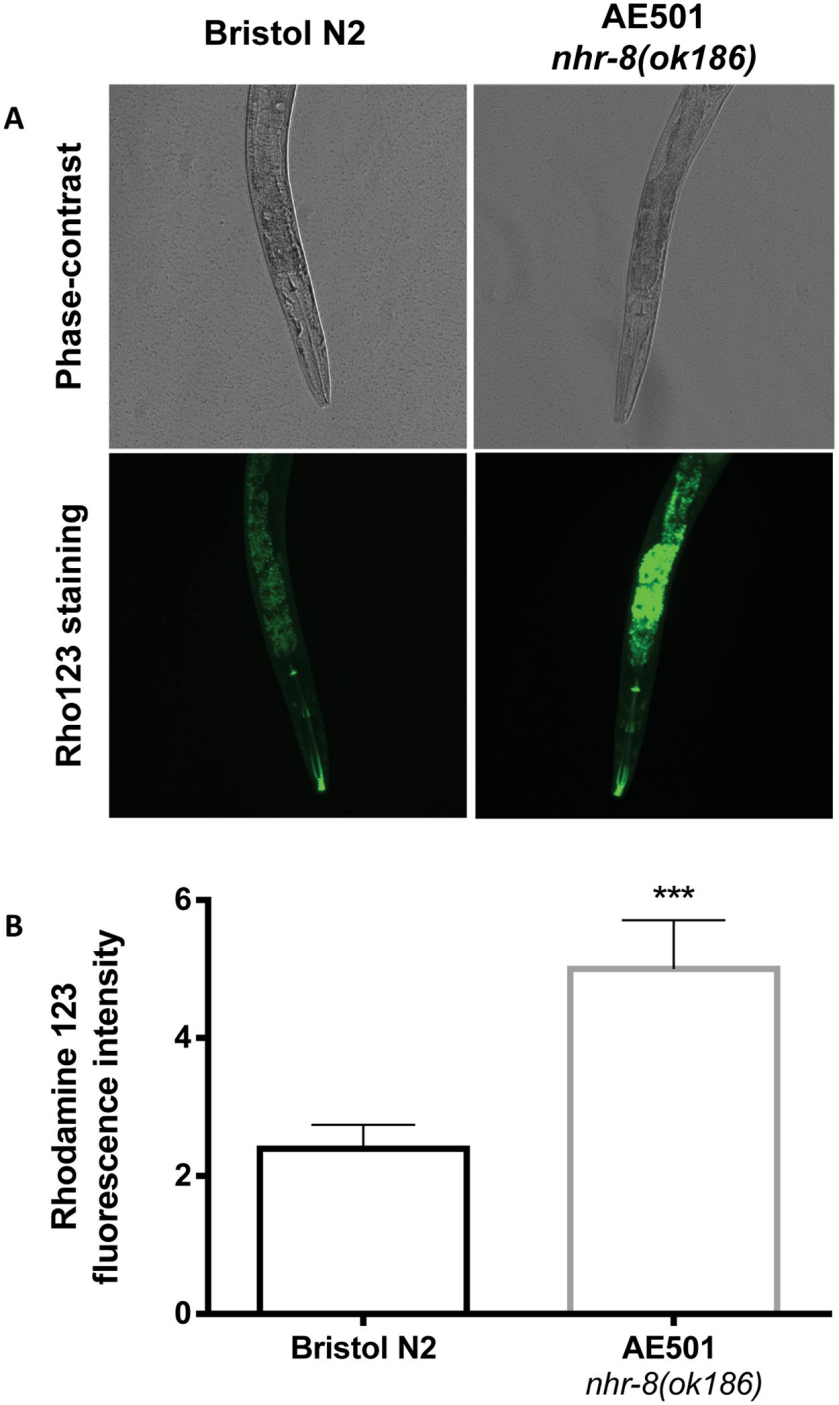
Effect of *nhr-8* loss on PGP function in *C*. *elegans*. PGP function was evaluated by measuring the accumulation of the fluorescent dye rhodamine 123 in *C*. *elegans*. Wild-type Bristol N2 and *nhr-8(ok186)* young adult worms were incubated with rho123 at 10 μM for 48 h at 21°C and then examined by fluorescence microscopy for fluorescence intensity quantification. (A) Representative micrographs of individual Bristol N2 and *nhr-8(ok186)* worms after a 48h incubation with rhodamine 123; (B) Quantification of fluorescence intensity in Bristol N2 and *nhr-8(ok186)* worms. Data are mean ± SEM from 30–40 worms per strain. *** p<0.001 vs wild-type.

Since several ABC transporters encoding genes with putative drug efflux function are repressed in *nhr-8(ok186)* mutants, we investigated the ABC-mediated transport activity by following the accumulation of the known mammalian PGP fluorescent substrate rhodamine 123 (rho123) into the worms. Accumulation of this xenobiotic was 2.1-fold higher in *nhr-8(ok186)* mutants than in wild-type *C*. *elegans* (Fig 5, p<0.001). These data show that the PGP-mediated transport of xenobiotics is reduced in *nhr-8(ok186)* mutants compared with wild-type worms and are in accordance with the role of NHR-8 in regulating genes involved in xenobiotic transport. Moreover, this provide a functional evidence of a link between *nhr-8* loss and altered drug distribution and elimination that underlies the observed shift in IVM sensitivity.

### *Pgp-6* is involved in IVM tolerance in *nhr-8(ok186)* mutants and IVM-resistant worms

Since the level of *pgp-6* expression shows the most pronounced inverse relationship with IVM susceptibility, we investigated the role of *pgp-6* in IVM tolerance of *C*. *elegans*. We first transformed the AE501(*nhr-8(ok186)*) strain with cDNA encoding *Cel-pgp-6* under the control of the strong constitutively active *Cel-eft-3* promoter. IVM efficacy was determined by monitoring the impact of a 20-min exposure to IVM on the pharyngeal pumping rate. No significant difference in the pump rate was observed between wild-type, AE501(*nhr-8(ok186)*) and AE501 expressing *Cel-pgp-6* worms without IVM exposure (4.5 ± 0.1, 4.3 ± 0.1, 4.2 ± 0.1 Hz, respectively, Table 3). Interestingly, exposure of transgenic *nhr-8*(ok186) worms transformed with *pgp-6* to IVM resulted in a rescue of the pharyngeal pumping frequency to wild-type level (2.4 ± 0.6 Hz; Table 3), revealing that *pgp-6* overexpression reverses the IVM hypersensitivity phenotype of *nhr-8* deficient animals.

**Table 3:** Effect of IVM on pharyngeal pumping activity of wild-type, *nhr-8* deficient and transgenic *C*. *elegans* strains.

	Pump frequency (Hz)		
	Untreated	IVM 0.1 μM	IVM ratio
**Bristol N2**	4.5 ± 0.1	2.1. ± 0.2 ^a^	2.4
**AE501 *nhr-8(ok186)***	4.4 ± 0.1	0.3 ± 01 ^a^^,^ ^b^	13.0
***Cel-nhr-8* in AE501**	4.5 ± 0.1	2.0 ± 0.1 ^a^	2.2
***Cel-pgp-6* in AE501**	4.2 ± 0.1	2.4 ± 0.2 ^a^	1.8
***Hco-nhr-8* in AE501**	4.6 ± 0.1	2.1 ± 0.2 ^a^	2.1

Pump frequency recorded with the Nemametrix ScreenChip system. Mean ± SEM; n = 30–90 worms/group.

IVM potency ratio as pump frequency with no treatment / pump frequency following exposure with IVM at 0.1 μM.

^a^ p<0.001 *vs* untreated worms;

^b^ p<0.001 *vs* wild-type Bristol N2.

**Fig 6:**
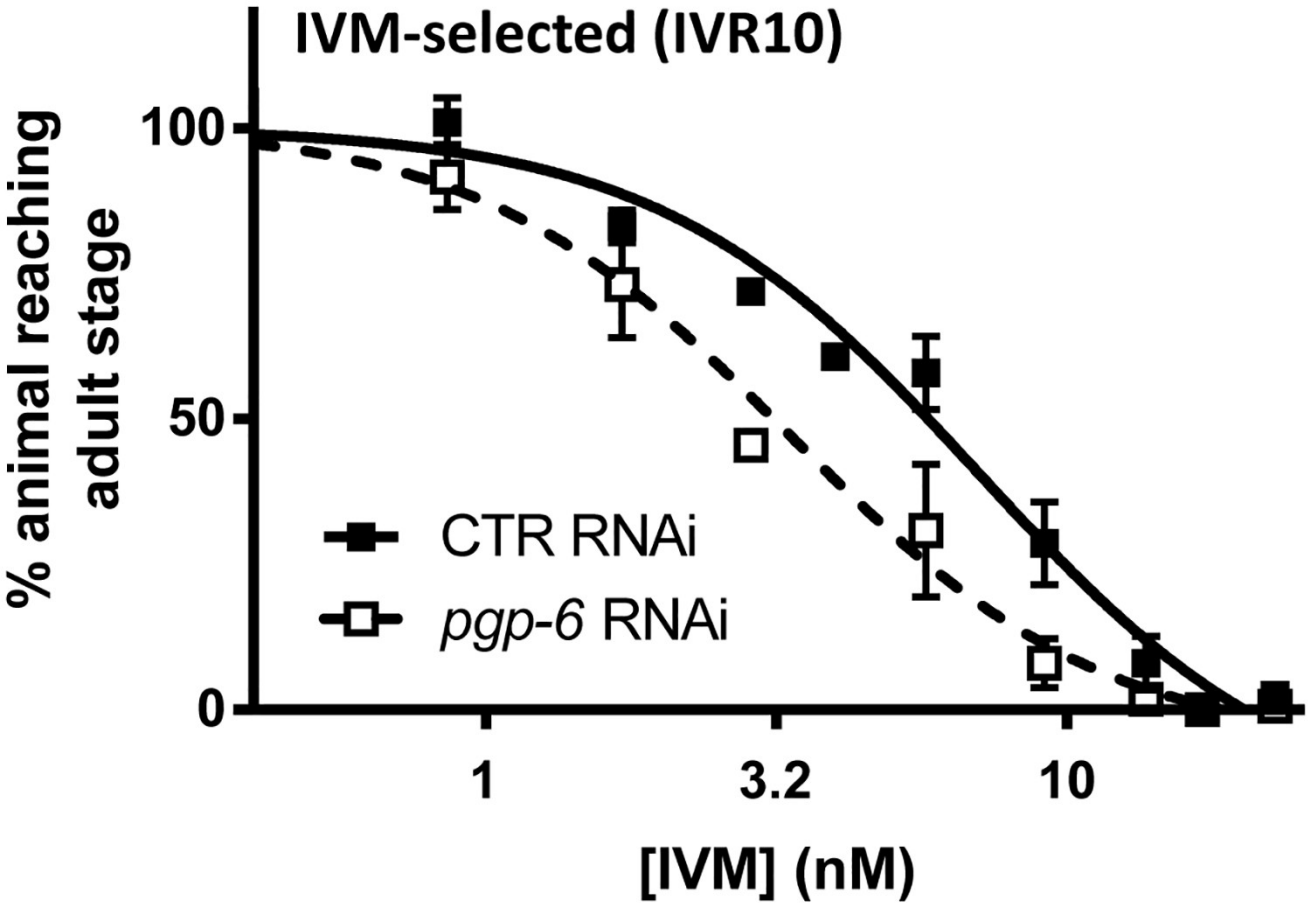
Effects of *pgp-6* silencing on susceptibility of IVM-resistant *C*. *elegans* (IVR10) to IVM in a larval development assay. Values for dose response curves to IVM of IVM-selected worms, fed on *pgp-6* or control RNAi represent the percentage of L1 reaching the young adult stage after 55 hours of incubation at 21°C within the presence of increasing doses of IVM. Data are mean ± SD from 3 independent experiments.

We then determined the effect of *pgp-6* silencing on the susceptibility to IVM of the IVM-resistant *C*. *elegans* strain (IVR10) using a larval development assay. Silencing of *pgp-6* induced a slight but significant increase of IVM efficacy in IVM-resistant *C*. *elegans*, as revealed by the shift to the left of the dose-response curve (Fig 6) and the lower EC_50_ measured in the *pgp-6* RNAi animals, compared with the control RNAi (p<0.05). As a result, *pgp-6* RNAi in IVM-selected strains were 2.1-fold more sensitive to IVM than their control RNAi counterparts. These results clearly show that *pgp-6* contributes to IVM tolerance in drug resistant *C*. *elegans*.

### *nhr-8* is required for development of tolerance against IVM in *C*. *elegans*

**Fig 7:**
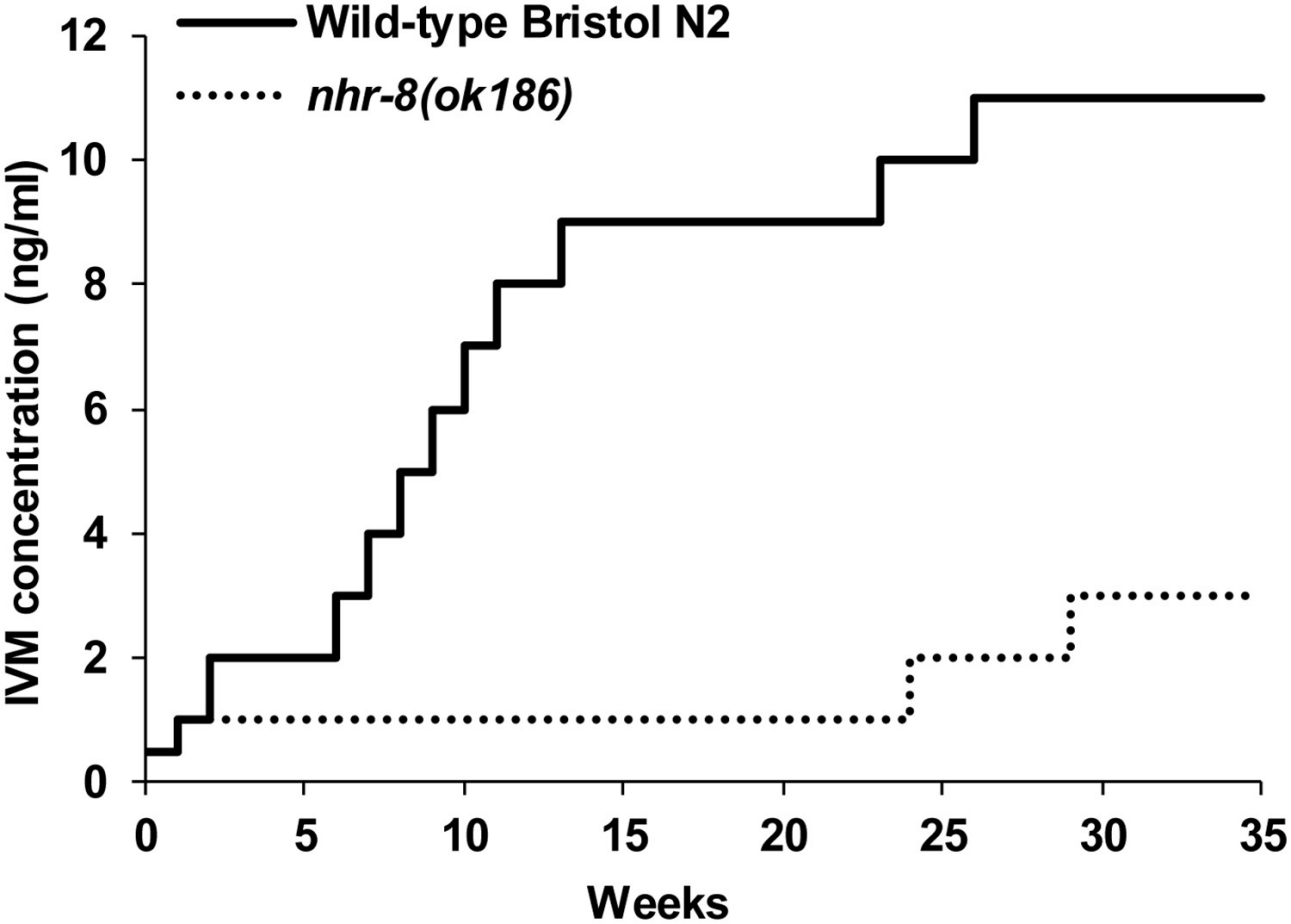
Time course of stepwise selection of IVM tolerance in wild-type and *nhr-8*-deficient *C*. *elegans* strains. Comparison between the development kinetics of acquired resistance to IVM in wild-type N2 Bristol (solid line) and *nhr-8*-*(ok186)* (dashed line) *C*. *elegans* strains following stepwise exposure to IVM. Worms, cultured on Nematode Growth Medium plates containing IVM, were transferred to NGM plates containing higher doses of MLs when able to survive and reproduce. The period of 35 weeks corresponds approximately to 60 generations for each strain. Data representative of three separate experiments.

We then investigate the role of *nhr-8* in the adaptation of *C*. *elegans* to IVM selection pressure. Wild-type Bristol N2 worms and *nhr-8* mutants were treated with sublethal doses of IVM and the dose was increased when the worms were able to survive and reproduce. The kinetics of adaptation of both *C*. *elegans* strains to IVM over a 35-week period show that wild-type and *nhr-8* mutant *C*. *elegans* differentially acquire tolerance to IVM (Fig 7). Wild-type *C*. *elegans* were able to rapidly acquire tolerance to IVM, as demonstrated earlier [15], whereas worms lacking *nhr-8* failed to develop the same level of tolerance to the drug after the same period of time. Indeed, after approximately 25 weeks, wild-type worms were able to survive on 9 ng/ml of IVM while *nhr-8*-deficient mutants were still difficult to cultivate on 1 ng/ml. This experimental adaptation study was repeated three times using equally sized populations of wild-type and *nhr-8* mutant *C*. *elegans*. These data show that loss of *nhr-8* postponed the acquisition of tolerance to IVM under drug selection pressure and reduced the level of acquired tolerance to the drug.

**Fig 8:**
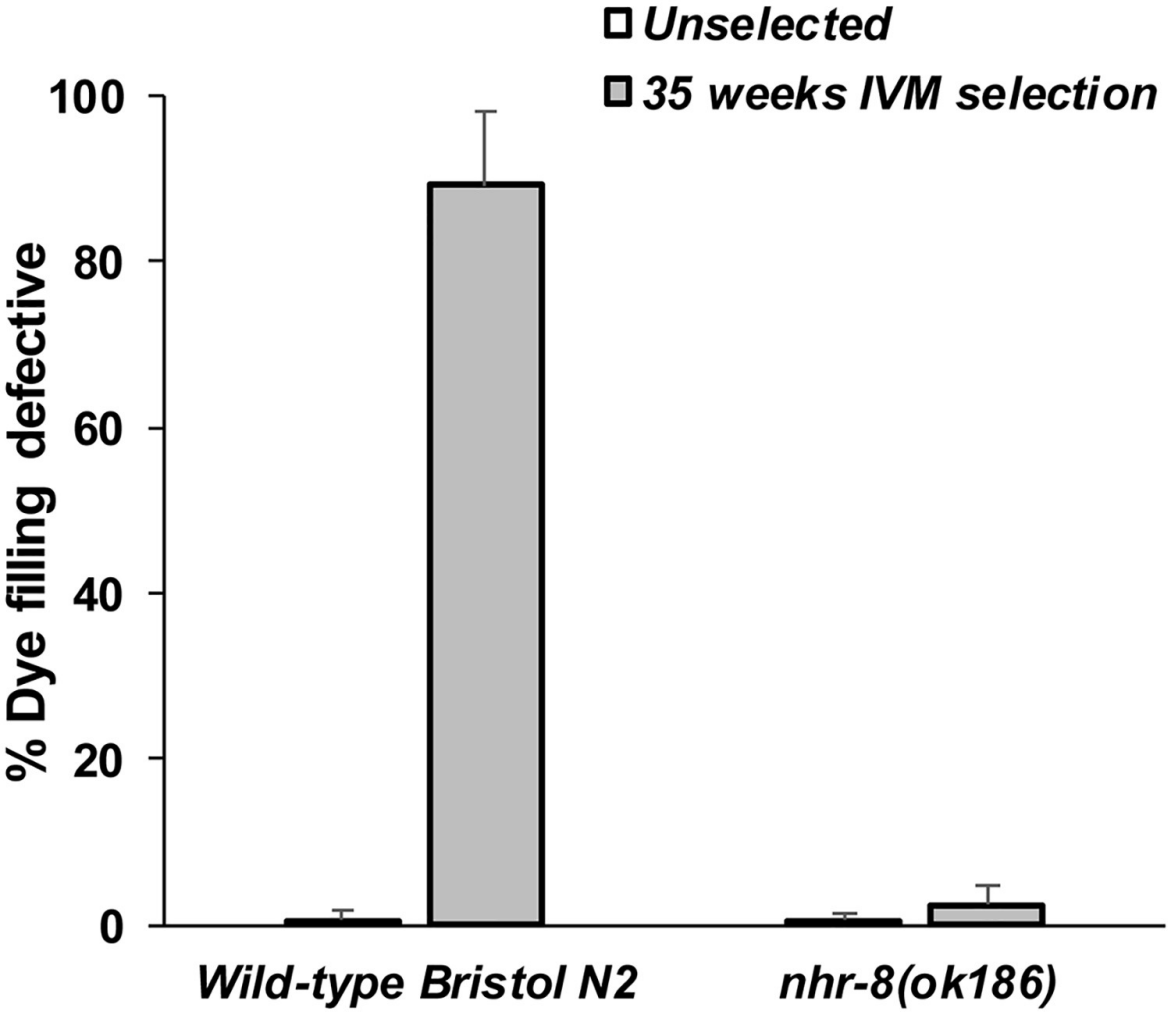
Percentage of *C*. *elegans* with dye-filling defective amphid neurons phenotype. Dye-filling of amphid neurons in the wild-type Bristol N2 strain and *nhr-8(ok186)* mutants was analysed before (unselected, white bar) and after a 35-weeks period of drug selection pressure with IVM (grey bar) (see Material & methods section for the drug selection process). Young adult worms were examined by fluorescence microscopy to visualize dye filling of the amphid dendrites after staining with the fluorescent dye DiIC12(3) (DiI) and scored for the dye filling (Dyf) defective phenotype (absence of red staining of the amphid neuron). Typical patterns of dye-filled amphidial dendrites from wild-type Bristol N2 worms and *nhr-8(ok186)* mutants are shown in S4 Fig.

A dye-filling defective phenotype in nematodes linked to defect in the integrity of amphidial neurons is associated with IVM resistance [35,36] and is a mechanism of acquired tolerance to MLs under drug selection pressure [15,37]. We therefore evaluated the dye-filling phenotype of wild-type and *nhr-8(ok186)* worms subjected to 35 weeks of IVM selection pressure. We confirm that the wild-type Bristol N2 strain displayed a normal morphology of amphid neurons with less than 1% of animals exhibiting a dye-filling defect, while IVM selection in a wild-type genetic background *C*. *elegans* led to a dye-filling-defective phenotype in more than 90% of the population (Fig 8). More interestingly, IVM drug pressure on worms lacking *nhr-8* failed in selecting for a dye-filling-defective phenotype. Even after 35 weeks of IVM selection, more than 97% of *nhr-8* mutants showed normal morphology of amphid neurons (Fig 8 & S4 Fig). In addition, the dye filling defective phenotype observed in IVM-resistant *C*. *elegans* (IVR10) was not restored when *nhr-8* was silenced by RNAi (S5 Fig). Altogether, these results show that the mechanism of acquired tolerance to IVM under selection pressure in *C*. *elegans* involving a defect in the integrity of chemosensory neurons depends on *nhr-8*.

### *Hco-nhr-8* of *H*. *contortus* is a functional ortholog of *C*. *elegans nhr-8*

Protein sequence alignment between Cel-NHR-8 and the orthologous protein from the parasitic nematode, *H*. *contortus* Hco-NHR-8 reveals a high identity between the two sequences. Compared with Cel-NHR-8, the DNA Binding Domain (DBD) of Hco-NHR-8 displays conserved cyteines that comprise a Zn coordination site in the DBD (S6A Fig). In addition, Ligand Biding Domains (LBD) from Cel-NHR-8 and Hco-NHR-8 showed conserved α helices (S6B Fig) and high similarity with 41.2% identity (Table 4). This supports that the function of Hco-NHR-8 could be conserved.

**Table 4:** Percentage of amino acid identity and similarity between full length, LBD and DBD sequence of NHR-8 from *C*. *elegans* and *H*. *contortus* determined after alignment with PAM 250 scoring matrix using LALIGN.

	Cel-NHR-8 and Hco-NHR-8 amino acid sequence	
	% identity	% similarity
Full length protein sequence	37.7	74.4
DNA Binding Domain (DBD)	76.1	98.6
Ligand Binding Domain (LBD)	41.2	82.0

In order to investigate the function of the *nhr-8* homolog of *H*. *contortus*, *C*. *elegans* was used as a heterologous expression system. Transgenic *C*. *elegans* lines expressing full-length of *Hco-nhr-8* under the *Cel-nhr-8* promoter were generated. Their frequency of pharyngeal pumping was analyzed after a 20-min exposure to 0.1 μM IVM and the effect was compared in wild-type, *nhr-8(ok186)* and *nhr-8(ok186) C*. *elegans* rescued with a transgene encoding *Cel-nhr-8* (Table 3). Pump frequency of untreated transgenic worms expressing *Hco-nhr-8* was equivalent to pump frequency of wild-type worms (4.5 ± 0.1 Hz *vs* 4.6 ± 0.1 Hz, Table 3). Importantly, the IVM hypersensitive phenotype of *nhr-8(ok186)* animals was totally abolished in transgenic animals expressing *Hco-nhr-8*. Their susceptibility to IVM was similar to that of wild-type worms or transgenic *nhr-8(ok186)* rescued with *Cel-nhr-8*, as revealed by the equivalent reduction in pump frequency after exposure with 0.1 μM IVM leading to a similar IVM potency ratio of about 2 (Table 3). Taken together, these results show that the transformation of AE501 *nhr-8(ok186)* with *Cel-nhr-*8 or *Hco-nhr-8* cDNA, resulted in a full rescue of the *nhr-8* mutant phenotype, demonstrating that *Hco-nhr-8* is a functional homologue of *Cel-nhr-8*.

### *Hco-nhr-8* silencing increases IVM susceptibility in *H*. *contortus*

**Fig 9:**
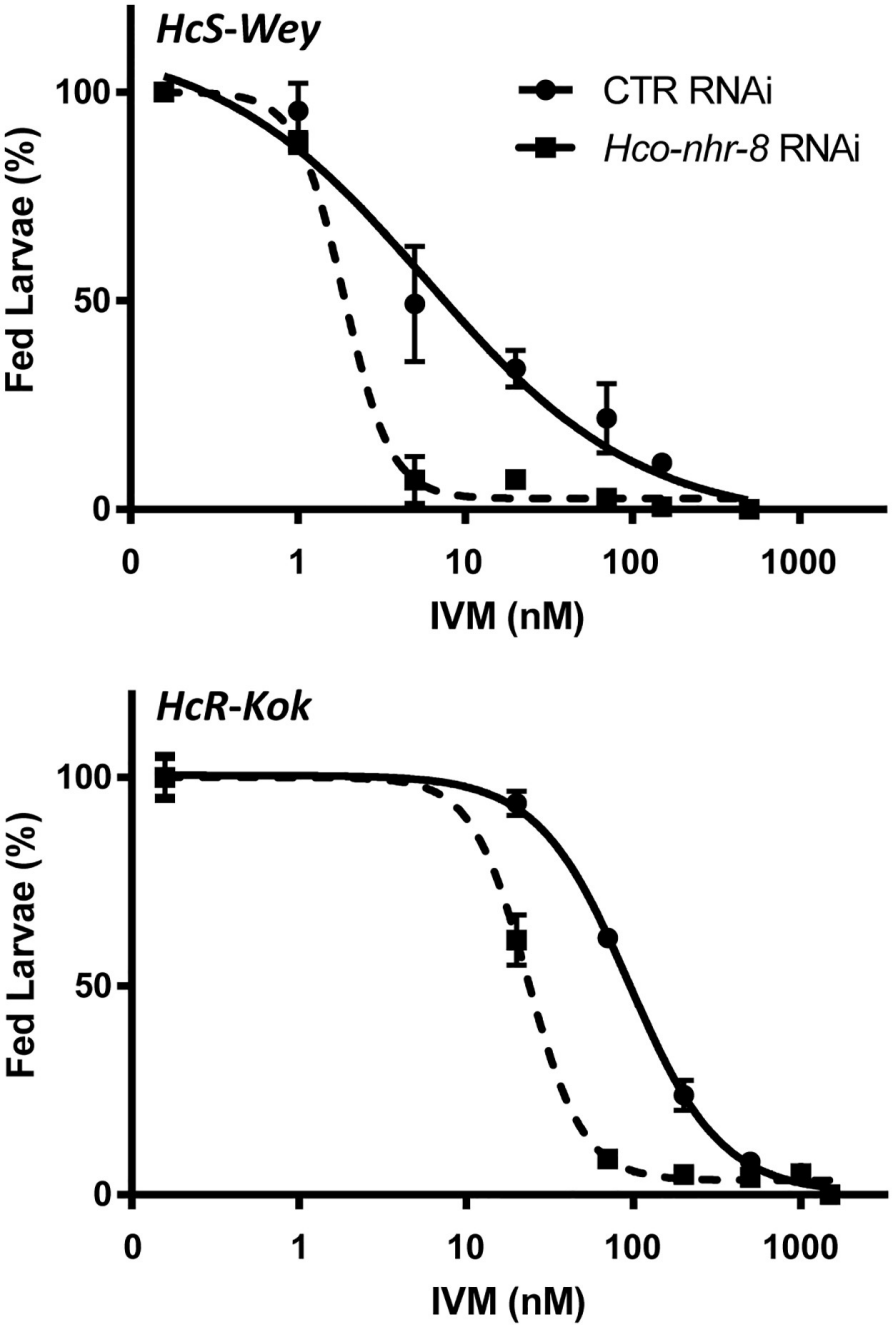
Impact of *Hco-nhr-8* silencing on IVM efficacy against susceptible (HcS-Wey) and resistant (HcR-Kok) *H*. *contortus* isolates. Larval feeding inhibition assay was performed by treating L2 larvae with increasing concentration of IVM, with or without silencing of *Hco-nhr-8* through RNAi technique using whether double stranded RNA specifically targeting *Hco-nhr-8* or non-target (*gfp*) siRNA as a control. The LFI_50_ estimates the concentration of IVM at which 50% of the L2 did not feed. Data are mean ± SEM from 3 independent experiments.

We then assessed the influence of *Hco-nhr-8* in IVM susceptibility in susceptible and resistant *Haemonchus contortus* isolates using gene-specific silencing by RNAi. The feeding of IVM resistant and sensitive *H*. *contortus* second-stage larvae (L2) was assessed using a range of IVM concentrations (1–1500 nM) in the presence of control or *Hco-nhr-8* RNAi. Dose-response curves for IVM on larval feeding inhibition on control RNAi and *Hco-nhr-8* RNAi allowed the calculation of LFI_50_s values (i.e. the concentration of IVM at which 50% of the L2 did not feed) for each isolate. Silencing of *Hco-nhr-8* increased IVM efficacy in both susceptible and resistant isolates, as revealed by a significant shift to the left (low LFI_50_) of the dose-response curves, compared with the control RNAi (Fig 9). LFI_50_s of IVM on *Hco-nhr-8* RNAi were significantly reduced compared with control from 5.9 ± 2.4 nM to 1.9 ± 0.2 nM (3.1 fold, p<0.05) and from 94.4 ± 6.1 nM to 23.2 ± 1.2 nM (4.1 fold, p<0.001), in susceptible and resistant isolate, respectively (Table 5). Altogether, these results clearly demonstrate that NHR-8 is able to modulate IVM efficacy in both susceptible and resistant *H*. *contortus* nematodes.

**Table 5:** Effect of *Hco-nhr-8* silencing on susceptibilities of *H*. *contortus* larvae to IVM.

	LFI_50_ IVM (nM)	
	HcS-Wey	HcR-Kok
Control RNAi	5.9 ± 2.4	94.4 ± 6.1
*Hco-nhr-8* RNAi	1.9 ± 0.2 *	23.2 ± 1.2 ***
R	3.1	4.1

LFI_50_ (concentration for 50% inhibition of the feeding).

R is the ratio of the LFI50 of IVM with control RNAi to the LFI_50_ of IVM with *Hco-nhr-8* RNAi.

*** p<0.001;

* p<0.05 vs Control RNAi. Data are mean ± S.D from 3 independent experiments.

## Discussion

Overexpression of genes of the xenobiotic detoxification network is a pleiotropic mechanism that contributes to drug resistance in pathogens. Overexpression of ABC efflux transporters occurring in response to IVM exposure in nematodes protects the target organism against anthelmintic toxicity and leads to drug resistance, certainly by increasing the elimination of the drug [38]. Moreover, biotransformation enzymes are also overexpressed in IVM-resistant *C*. *elegans* [10,15]. Regulation of drug detoxification genes in mammals occurs mainly by transcriptional events through activation of specific nuclear receptors named “xenosensors”, but very little information exists on the mechanisms by which these genes are regulated in nematodes. In that context, the aim of this study was to evaluate the possible involvement of transcription factors in IVM tolerance and in the regulation of genes from the detoxification network in nematodes.

We used *C*. *elegans* strains with loss-of-function in some specific transcriptional regulators to identify their role as modulators of IVM sensitivity. Loss of the specific regulator NHR-8 rendered nematodes more susceptible to IVM than the wild-type. By contrast, neither loss of NHR-48 nor DAF-12, which are closely related in sequence to NHR-8 [39], altered the susceptibility of the worms to IVM. IVM was more effective in *nhr-8* mutants than in wild-type in inhibiting larval development from L1 to the young adult stage (mid-term exposure of 55h), and in blocking the pharyngeal pumping (short-term exposure of 20 min). This demonstrates that *nhr-8* mutants exhibit an intrinsic hypersensitivity phenotype to IVM. It has to be noted that mutants with the weak allele ok(186) displayed no apparent development defect or delay, as reported previously [30,40], thereby bypassing potentially confounding effects of *nhr-8* deficiency on worm development. Importantly, silencing of *nhr-8* by RNA-mediated interference (RNAi) in IVM-resistant *C*. *elegans* strains also resulted in increased IVM efficacy. Altogether, these data indicate that a reduction of *nhr-8* function in both susceptible and resistant *C*. *elegans* results in hypersensitivity to IVM.

Interestingly, NHR-8 is primarily expressed in the intestine, which constitutes the major metabolic organ of *C*. *elegans* [31], and it has been specifically associated with a general response to xenobiotics [30]. Indeed, *nhr-8(ok186)* loss-of-function mutants used in this study are hypersensitive to the toxins colchicine and chloroquine, and display low expression of some xenobiotic detoxification genes [29,30,41]. Moreover, NHR-8 is closely related to the mammalian xenobiotic receptors CAR (constitutive androstane receptor), PXR (pregnane X receptor) and VDR (vitamin D receptor) [28,30], which play major roles in regulating the xenobiotic response [42–45]. Similarly, the NHR-8-related receptor in *Drosophila*, DHR96 [45] is involved in susceptibility to xenobiotics (e.g. phenobarbital, DDT and permethrin) and is required for induction of detoxification genes by xenobiotics [46,47].

We thus hypothesize that the expression of genes encoding proteins that play a role in biotransformation and elimination of IVM may be impaired in *nhr-8*-deficient worms. This includes particularly PGPs of *C*. *elegans* [48,49] and parasitic nematodes [50–53]. Interestingly, loss of *nhr-8* increases the susceptibility of *C*. *elegans* to IVM to an extent comparable to what was observed after inhibition of PGPs function [15,54]. In an initial probe of potential NHR-8 target genes, we assayed the expression of more than 50 *C*. *elegans* genes that belong to the detoxification network and are inducible by xenobiotics [27]. The expression of 18 genes encoding ABC efflux transporters, CYPs and GSTs was constitutively down-regulated in *nhr-8(ok186)* compared with wild-type worms. Strikingly, 12 of these genes are also constitutively overexpressed in IVM-resistant *C*. *elegans*, such as *pgp-1*, *pgp-3*, *pgp-6*, *pgp-9*, *pgp-13* and *mrp-6* [15]. Interestingly, *Pgp-6* was the most highly inversely regulated gene between the IVM-hypersensitive *nhr-8* loss-of-function mutants (4-fold underexpressed) and the IVM-tolerant strains (5-fold overexpressed [15]). Since *pgp-6 and pgp-13* are expressed in the amphids and can protect *C*. *elegans* from ML toxicity [48,54], their overexpression could help in protecting nematodes from the effects of MLs on extrapharyngeal neurons associated with the amphids. In addition, *pgp-9*, which also showed inverse regulation between tolerant and hypersensitive worms, is involved in IVM susceptibility of *C*. *elegans* [54] and is overexpressed in ML-resistant *H*. *contortus* [14] and *Teladorsagia circumcincta* [55] isolates. Therefore, *pgp-*6, *pgp-9* and *pgp-13* are likely to be important IVM tolerance-associated gene. Finally, less pronounced, but still significant, inversed regulation of *pgp-1* and *pgp-3* expression is consistent with their protective role against heavy metals [56,57] and IVM [54] in *C*. *elegans*. Loss of *pgp* or inhibition of PGP function are associated with higher IVM efficacy in *C*. *elegans* and parasitic nematodes [38,54]. Our data further demonstrate that *nhr-8* loss in *C*. *elegans* is associated with an increased accumulation of PGP substrate in adult worms, showing that *nhr-8* loss induces a downregulation in gene expression of some PGPs and a concomitant decrease in PGP function. This supports the hypothesis that *nhr-8* plays a role in the tolerance of worms against IVM by regulating *pgps* gene expression and thereby drug transport, in agreement with the correlation between ABC efflux transporter expression and ML resistance previously reported in nematodes [58,59].

Because *pgp-6* was the most highly inversely regulated gene between *nhr-8* loss-of-function mutants and the IVM-resistant strain, we further evaluate the particular role of *pgp-6* in susceptibility to IVM. Transformation of AE501 *nhr-8(ok186)* with *pgp-6* cDNA, resulted in a return to a wild-type IVM tolerance phenotype of the IVM hypersensitive *nhr-8* mutants, while silencing of *pgp-6* in ML-resistant *C*. *elegans* resulted in an increase in IVM efficacy. These data demonstrated that the IVM hypersensitivity phenotype of *nhr-8(ok186)* mutants is due, at least partly, to a downregulation of *pgp-6* linked to the loss of NHR-8 function, and highlight the role of *pgp-6* in IVM sensitivity. However, it has to be kept in mind that *pgp-6* rescue experiments by micro-injection of the pgp-6 cDNA likely results in many copies of the injected construct in extrachromosomal arrays and over-expression of the gene. In addition, *nhr-8(ok186)* mutants harbor a down-regulation of 5 *pgp* genes other that *pgp-6* (*pgp-1*, *pgp-3*, *pgp-4*, *pgp-9* and *pgp-13*) and each individual PGP from *C*. *elegans* can play a role in IVM efficacy [54]. Therefore, we cannot conclude on the relative contribution of *pgp-6* in IVM efficacy, and it is likely that some other PGPs also contribute to the tolerance to IVM in our model. We expect that NHR-8, by regulating concomitantly the expression level of several *pgp* and other detoxification genes, affects IVM efficacy in a more pronounced manner than any single NHR-8-regulated gene.

Our transcriptomic analysis also showed specific modulations of *pmp-4* and *pmp-5*, orthologs of the human peroxisomal importers of fatty acids ABCD2 and ABCD4, suggesting a role of lipid transporters of *C*. *elegans* in IVM susceptibility. Moreover, *cyp14A*, *cyp35A* and *cyp37B* isoforms were constitutively and inversely regulated in *nhr-8(ok186)* and IVM-resistant worms. In line with this, members of the *C*. *elegans cyp14A* and *cyp35A* subfamily respond to a variety of xenobiotic stressors [27,60–62]. In addition, the *cyp14*, *cyp33*, *cyp34*, and *cyp35* families in *C*. *elegans* are orthologs of mammalian xenobiotic inducible genes *CYP1A2* [61] and the *CYP2* family [63], which are both induced by IVM [21–23]. Since the mammalian CYP genes are involved in IVM metabolism [64], these results suggest that their potential role in drug removal in the worm can occur in response to IVM exposure in an *nhr-8*-dependent manner. Moreover, in *C*. *elegans*, the closest CYPs to all of the clustered *H*. *contortus* parasite genes are CYP14 family members [65], and may therefore represent candidate IVM metabolizing genes in the parasite.

Finally, *gst-4* and *gst-10* were inversely regulated in IVM-tolerant and IVM-hypersensitive *nhr-8(ok186)* worms. These genes encode glutathione S-transferases that are essential detoxifying enzymes involved in reducing reactive oxygen species (ROS) and extending stress resistance, especially in the context of paraquat exposure [66,67]. Since MLs are known to induce oxidative stress and decrease antioxidant enzyme activities in mammals [68] and in birds [69], *gst-4* and *gst-10* could also be IVM tolerance-associated genes.

This shows that NHR-8 can regulate the transcription of genes associated with xenobiotic metabolism and transport, and loss of *nhr-8* may lead to a decrease in IVM elimination, making the worms hypersensitive to the drug. Moreover, the inversely regulated gene expression of several genes encoding ABC transporters, cytochromes and transferases in IVM-tolerant and *nhr-8* mutants further supports the hypothesis that IVM can regulate genes involved in drug detoxification in an *nhr-8*-dependent manner. Nevertheless, to assess the direct link between *nhr-8*-regulated genes and IVM tolerance, further experiments have to be envisaged in *C*. *elegans* by expressing genes of interest and assessing changes in IVM sensitivity in wild-type and *nhr-8* mutant worms.

The ability of worms to adapt to drugs remains the greatest impediment to sustainable efficacy of anthelmintics. Exposure of parasites to subtherapeutic drug concentrations favor the development of resistance [70] and lead to selection for IVM resistance within only few generations [10,15,71]. We recently demonstrated that the xenobiotic detoxification network is modulated after acquisition of ML tolerance through drug selection pressure [15]. Therefore, we performed a comparative experimental adaptation study of *C*. *elegans* by passaging wild-type N2 Bristol and *nhr-8*-deficient worms through more than 60 generations at gradually increasing IVM doses. Interestingly, while the experimental evolution of wild-type *C*. *elegans* populations in IVM-enriched environment resulted in adaptation and development of tolerance to this environment in a very few generations (Fig 7), *nhr*-8-deficient animals lost such adaptive capacity. This indicates that *nhr-8* function is essential not only for IVM susceptibility, but also for the dynamic of development of tolerance to IVM in worms.

Notably, ML resistance in nematodes has a multigenic basis [6]. In our study, the expression of none of the standard genes that are known to be associated with IVM-resistance, such as *glc-1*, *glc-2*, *avr-14*, *avr-15*, *unc-7*, *unc-9* [35] and *dyf-7* [37]s, is modulated by loss of *nhr-8* (S3 Fig). However, mutations in genes involved in the development of amphidial neurons, leading to a defect in amphid neuron integrity, are associated with tolerance to MLs [15,35,37]. In this study, when subjected to a long-term IVM selection pressure, *nhr-8(ok186)* mutants showed normal morphology of amphids neurons. This reveals that the mechanism of acquired tolerance to MLs involving a defect in the integrity of chemosensory neurons depends of *nhr-8*.

Taken together, these results strongly suggest that the acquisition of IVM resistance is a pharmacokinetic-mediated process. Indeed, we have previously reported that adaptation to drug pressure with MLs led to constitutively elevated expression levels of phase I, phase II and phase III detoxification genes [15]. In addition, several nematode species such as *C*. *elegans* [10], *H*. *contortus* [14], *T*. *circumcinta* [72], and *Brugia malayi* [11] were shown to rapidly upregulate protective pathways in response to IVM. This is consistent evidence that induction of nematode xenobiotic detoxification system during drug selection pressure is an important requirement for IVM-induced tolerance in nematodes. Here we show that NHR-8, a transcription factor that controls the expression of detoxification genes, is an important mediator in this process. NHR-8 may allow the enhancement of the xenobiotic detoxification system upon exposure to repeated-sublethal doses of IVM. Then, the increased active efflux of the drug, together with its enhanced deactivation via biotransformation, diminishes drug efficacy, thus facilitating the survival of some individual worms to the anthelmintic therapy. Repetitive outliving of these “less-sensitive” worms allows a gradual selection, which culminates in the development of a resistant strain. Loss of *nhr-8* avoids upregulation of the xenobiotic detoxification system under drug selection, thus preventing the appearance of tolerant worms and the possibility of accumulation of crucial mutations leading to resistance among them. Whether IVM is able to directly or indirectly activate NHR-8 remains to be investigated.

Several parasitic nematode species, including *H*. *contortu*s and *B*. *malayi*, harbor *nhr-8* orthologs [31]. In this study, we were able to restore wild-type IVM sensitivity in *nhr-8(ok186)* mutants by expressing cDNAs derived from *Hco-nhr-8*, the ortholog of *nhr-8* from *H*. *contortus*. Above all, *in vitro* silencing of *Hco-nhr-8* in both susceptible and resistant *H*. *contortus* isolates resulted in an increased susceptibility to IVM, demonstrating that NHR-8 function is conserved between *C*. *elegans* and *H*. *contortus*, and suggesting that similar mechanisms could occur in other parasitic nematodes. In addition, this highlights that *C*. *elegans* can be a good model to study genes possibly involved in drug tolerance, especially concerning macrocyclic lactones.

We hypothesize that in nematodes, *nhr-8* contributes to the development of IVM tolerance by increasing the transcription levels of xenobiotic detoxification genes in IVM-exposed worms, leading to modulation of drug concentrations in worm. Interestingly, a recent study analyzing candidate resistance-associated genes with signature of anthelmintic selection in *H*. *contortus* isolates identified efflux pump and transcriptional regulation factors as genes possibly involved in resistance to IVM [13].

Altogether, the present study shows that NHR-8 simultaneously regulates multiple genes putatively involved in IVM detoxification by *C*. *elegans*. This nuclear receptor therefore represents a strategic target to substantially improve IVM efficacy. Agents that specifically decrease or eliminate the activity of NHR-8, such as high-affinity or high-specificity antagonists of the NHR-8 protein, should be envisaged as co-drugs to be administered in combination with IVM, and possibly other current anthelmintics, in order to (i) increase the efficacy of the drug against isolates with acquired tolerance to the drug, and (ii) limit the development of drug resistance thereby increasing the useful life of current and future anthelmintics.

Finally, it is interesting to note that NHR-8 controls metabolic pathways important in life history traits that are crucial for the fitness of the worms. These include lifespan, fecundity, growth and reproduction as well as lipid homeostasis. Indeed, by regulating cholesterol balance and production of dafrachronic acid, a bile acid-like steroid that controls longevity, NHR-8 is proposed to be the *C*. *elegans* functional homolog of the mammalian sterol-sensing receptors LXR and FXR [31,32,73]. Since we have shown that NHR-8 is involved in IVM tolerance, it is reasonable to speculate that NHR-8 will be at the center of the interplay between ML resistance and metabolic changes related to the fitness cost in nematodes. In support with this hypothesis, besides bile acids, IVM and other MLs are partial agonists of FXR [25,74]. One can therefore assume that IVM or a derivative may bind and modulate NHR-8.

These results shed light on an important and specific role of *nhr-8* in IVM tolerance in nematodes. NHR-8 was shown to regulate genes encoding xenobiotic detoxification pathways, including several PGPs, that were also identified as IVM tolerance-associated genes. Even if future research is needed to identify other enzymes or transporters that specifically contribute to NHR-8-dependent IVM tolerance in *C*. *elegans*, targeting NHR-8 provides an innovative strategy that can be exploited to improve anthelmintic efficacy and delay the onset of resistance to these drugs in parasitic nematodes.

## Materials and methods

### Materials

IVM (IVM), dimethylsulfoxide (DMSO), sodium hypochlorite, cholesterol, tetracyclin, ampicillin, isopropyl-β-D-thiogalactoside (IPTG), serotonin (5-hydroxytryptamine, 5HT), rhodamine 123 and fluorescein 5-isothiocyanate (FITC) were purchased from Aldrich (Sigma, Aldrich Chimie, St Quentin Fallavier, France). Moxidectin (MOX) was a generous gift from Fort Dodge International (Fort Dodge, IA). Culture plates were supplied by Corning (Borre, France). For all experiments, IVM was dissolved in DMSO and the maximal concentration of DMSO was 0.5% in all assays.

### *C*. *elegans* nematode strains and cultivation conditions

Wild-type *C*. *elegans* Bristol strain N2 and the mutant strains AE501, *nhr-8(ok186)*; AA107, *nhr-48(ok178)*; DR20, *daf-12(m20)* as well as the OP50 and HT115(DE3) *Escherichia coli* strains were obtained from the *Caenorhabditis* Genetics Center (CGC, University of Minnesota, Minnesota, Minneapolis, MN, USA). AA968 *nhr-8(hd117)* and *AA1788 nhr-8(tm1800)* were a generous gift from Pr. Adam Antebi. IVR10 strain, selected from the wild-type Bristol strain N2 with IVM and phenotypically resistant to IVM was kindly provided by Dr C. E. James [10]. A MOX-selected strain, selected from the wild-type Bristol strain N2 with MOX, was described previously [15].

All strains were cultured and handled (unless otherwise stated) according to the procedures described previously [75]. Briefly, nematodes were cultured at 21°C on Nematode Growth Medium (NGM) agar plates (1.7% bacto agar, 0.2% bactopeptone, 50 mM NaCl, 5 mg/L Cholesterol, 1 mM CaCl_2_, 1 mM MgSO_4_, and 25 mM KPO_4_ Buffer) seeded with *Escherichia coli* strain OP50 as a food source. ML-containing NGM plates were prepared as follows: stock solutions of IVM and MOX in DMSO were diluted in NGM at the adequate concentration before pouring plates. IVM-selected strain (IVR10) was cultured on NGM plates containing 11.4 nM (10 ng/ml) of IVM and MOX-selected strain was cultured on NGM plates containing 4.6 nM (3 ng/ml) of MOX, as previously described [15].

Nematodes were synchronized through egg preparation with sodium hypochlorite. Briefly, an asynchronous population with majority of gravid adults and eggs was collected by washing the bottom of the NGM plates with M9 buffer (3 g KH_2_PO_4_, 6g Na_2_HPO_4_, 5 g NaCl, 0.25 g MgSO_4_ 7H_2_O in 1 l water) and centrifuged at 1200g for 1 minute. All larval stages except eggs were lysed with a bleaching mixture (5 M NaOH and 1% hypochloride). Third washes of M9 were done to remove the toxic bleaching mixture. *C*. *elegans* eggs were then hatched overnight at 21°C in M9 solution without bacteria to obtain a synchronized population of first-stage larvae (L1).

### Parasite isolates

The two *H*. *contortus* isolates tested were: (i) Weybridge (*HcS*-*WB*, *UK)*: a line susceptible to all anthelmintics with no history of exposure to MLs or other anthelmintics; (ii) Kokstad (*HcR-KOK*): a line resistant against the three main anthelmintic classes, i.e. levamisole, MLs and benzimidazole [76], originally obtained from a farm in South Africa and maintained in the INRA laboratory since 2000. Each isolate was individually passaged every 2 months in a 3-month-old sheep (préalpes breed) through infection with 6000 infective larvae (L3). Sheep carrying the resistant isolate of *H*. *contortus* were treated with IVM (0.2 mg/kg) at 35 day post infection.

### Larval development assay (LDA) on *C*. *elegans* strains

This assay measures the potency of anthelmintics to inhibit the development of *C*. *elegans* from eggs to the young adult stage. Approximately 30 synchronized L1 larvae were added in every well of a 12-well plate poured with NGM containing increasing concentrations of compound of interest and seeded with OP50 bacteria. DMSO was used as control with a maximal concentration of 0.3%. At this concentration, no harmful effects of the vehicle on *C*. *elegans* were observed. Plates were then incubated at 21°C in the dark for a period of 52–55 h in which L1 of the negative control were developed into late L4 /young adult worms. L1, L2 and L3 were scored as inhibited in their development and the late L4 and young adult worms were classified as developed. Development was calculated as a percentage of late L4 and young adults in the presence of compounds of interest normalized to the untreated control. Every concentration was set-up in triplicate and all experiments were independently replicated at least five times. Curve fitting for the larval development assay (sigmoidal dose-response curve with variable slope) was performed with the Prism 6 software package (GraphPad, SanDiego, CA, USA). This allowed calculation of EC_50_ values, i.e., the concentrations at which 50% of the animals fail to reach the young adult stage.

### RNA interference of *Cel-nhr-8* in *C*. *elegans*

RNA interference (RNAi) was conducted by providing a food source of HT115 bacteria transformed with L4440 vector that produces double-stranded RNA against the targeted gene. HT115 bacteria clones expressing *nhr-8* RNAi or the empty vector as control from the Ahringer RNAi library were grown for 8 h at 37°C in LB medium containing ampicillin (50 μg/ml), then seeded on NGM plates supplemented with carbenicilin (25 μg/ml) and IPTG (1 mM) to induce RNAi expression overnight. For larval development assay, increasing concentrations of IVM were added to NGM while DMSO was used as control. Synchronized and starved L1 larvae from both IVM-selected (IVR10) and MOX-selected strains were seeded on to the NGM plates with control RNAi or *nhr-8* RNAi. The worms were allowed to grow on the RNAi plates until the young adult stage. The extent of knockdown of *nhr-8* mRNA was determined by qRT-PCR (S2 Fig).

### Development of acquired tolerance to IVM in *C*. *elegans* wild-type Bristol N2 and *nhr-8(ok186)* mutants

Culture conditions for the development of ML-resistant *C*. *elegans* strains following stepwise exposure to MLs were previously described [10,15]. Briefly, at week 0, wild-type and *nhr-8*(*ok186*) worm populations were transferred to NGM plates containing 0.5 ng/ml of IVM. This concentration, determined in a preliminary assay, corresponds to the highest concentration allowing 100% of development to the adult stage. Twice a week, worms were transferred onto new NGM plates. When worms survived and reproduced, they were transferred onto plates containing higher doses of MLs. The IVM concentrations used to create IVM-selected strains from wild-type and *nhr-8(ok186)* genetic background were 0.5, 1, 2, 3, 4, 5, 6, 7, 8, 9 and 10 ng/ml. After 35 weeks, wild-type worms were able to survive on 10 ng/ml of IVM, whereas *nhr-8(ok186)* worms were only able to survive on 3 ng/ml of IVM.

### *C*. *elegans* dye filling assay

Amphidial dendrites of *C*. *elegans* in wild-type Bristol N2 and *nhr-8(ok186)* were visualized by DiI staining of amphids, before and after 35 weeks of drug selection pressure with IVM. Worms were synchronized at the young adult stage and larvae were incubated in a dye solution containing 10 ng/ml of DiIC_12_(3) (1,1'-Didodecyl-3,3,3',3'-Tetramethylindocarbocyanine Perchlorate) in M9 with gentle shaking for 2 h at 21°C. After a recovering period of 2 h on NGM plates, worms were paralyzed using levamisole (40 mM) and dye-filled L4s were observed using a Nikon Eclipse 50i microscope equipped with a Luca S camera and analyzed using Nikon ACT-1 software. Animals were scored for the red staining of the amphids neurons and expressed as percentage of animals with a dye-filling defective phenotype.

### *H*. *contortus* L2 cultures and RNA silencing technique

*Haemonchus contortus* eggs were extracted from fresh fecal material using the standard procedure described by Rossanigo and Gruner [77]. L2 Larvae used in gene silencing assays were obtained from *in vitro* culture of eggs. Approximately 2000–3000 eggs/mL were cultured horizontally in tissue culture flasks at 20°C in a nutritive medium (0.1 mL per mL of culture of 1X Earle’s balanced salt solution (Sigma-Aldrich) and 0.5% (w/v) of yeast extract). The experimental procedures for feeding and gene silencing assays were performed on 4000 (3 day-old) L2 larvae in a final volume of 500 μl in 5 ml tubes, as previously described [78]. 1 μM non-target (*gfp*) siRNA labeled with the Alexa 594 (Eurogentec, Belgium) was added to the culture medium. After 2 hours of incubation under gentle agitation at 20°C, L2 larvae were washed three times with water and ingestion was monitored under fluorescence microscopy. 20 μl of a 20 μM siRNA solution in water was added. Double stranded RNA specifically targeting *Hco-nhr-8* were synthesized by Eurogentec (Belgium). *Hco-nhr-8* dsRNA sequences were: forward: CCAACAAAUUCGAGCAAAUdTdT and reverse AUUUGCUCGAAUUUGUUGGdTdT. All tubes were gently shaken horizontally for 48 h at 20°C before a Larval Feeding Inhibition assay was performed. The absence of toxicity was confirmed by comparing the viability of dsRNA treated vs untreated L2 larva after 48 h of incubation. The relevance of using the L2 stage was confirmed with preliminary sensitivity assays showing that L2 larvae are highly sensitive to IVM and with RT-qPCR experiments confirming the expression of *Hco-nhr-8* in this developmental stage (S8 Fig).

### Larval feeding inhibition assay (LFIA) on *H*. *contortus*

The larval feeding inhibition assay consists in the study of the reduction of food ingestion (due to pharyngeal muscles paralysis) by second-stage larvae (L2) incubated in serial dilutions of IVM, as previously described [19]. The percentage of larvae fed is determined for each concentration by examination of the larvae’s intestine, and the dose of larval feeding inhibition 50 (LFI_50_) (i.e., the concentration of IVM required to inhibit the ingestion in 50% of the L2) is calculated.

After 48 h of incubation with dsRNA at 20°C, IVM was added at the desired final concentration. After 2 h of incubation in the presence of the drug at 20°C, 10 μl of an FITC solution (2 mg/ml in PBS) were added to the 500 μL, and the cultures were reincubated at 20°C overnight. Following this incubation, larvae were sedimented and transferred onto a glass slide for counting. Observation of intestinal fluorescence was carried out at a magnification of x100 through a fluorescence inverted microscope fitted with a UV blue range filter (495 nm). At least 200 larvae were counted in each sample and larvae with FITC visible in the larval oesophagus and throughout the gastrointestinal tract were considered to be fed, while larvae lacking FITC labelling in the gut or with green fluorescence only around the buccal opening were considered unfed. A least two samples were analyzed for control and per drug concentration and each assay included two independent replicates. Control samples provided a qualitative measure of larval viability. Logarithm of doses against percentage of fed larvae was plotted and the LFI_50_ for IVM in the presence of control RNAi or *nhr-8* RNAi was calculated using the Prism 6 software package (GraphPad, SanDiego, CA, USA).

### Total RNA isolation and RT-PCR analysis

#### Isolation of RNA and cDNA synthesis

Changes in gene expression profiles in AE501 *nhr-8(ok186) C*. *elegans* strain compared to the wild-type Bristol N2 strain were analyzed using real-time qRT-PCR. Synchronized L1 larvae were added to control NGM plates (1000 L1 larvae were distributed onto 10 cm NGM/OP50 plates). After 55 h of incubation at 21°C, synchronized young adults were collected using M9 buffer. After five washes with M9, sedimented worms were added to 1 mL TRIzol reagent (Invitrogen, Cergy Pontoise, France), frozen in liquid nitrogen and stored at -80°C. Frozen samples were then thawed, homogenized twice for 10 s at 6 m.s^−1^ in a FastPrep-24 instrument (MP-Biomedicals, NY, USA) and total RNA was extracted according to the manufacturer’s instructions. Total RNA was quantified using a NanoDrop ND-1000 spectrophotometer (NanoDrop Technologies Inc., Wilmington, DE, USA). RNA purity was checked by measurement of the A_260/280 nm_ ratio, which was routinely in the range of 1.8–2.0, and RNA quality control was carried out using an Agilent 2100 Bioanalyser (Agilent Technologies, Waldbronn, Germany). cDNA was synthesized from 2 μg of total RNA using the High-Capacity cDNA Reverse Transcription kit (Applied Biosystems—Life Technologies, Courtaboeuf, France).

#### Single worm RNA analysis

Quantification of expression of the transgene in transgenic *C*. *elegans* strains, RNA extraction, cDNA synthesis and RT-qPCR was performed on individual *C*. *elegans* using a protocol adapted from Ly et al. [79]. A single worm was picked from an NGM plate and transferred into 1 μl of worm lysis buffer (5 mM Tris pH 8.0, 0.5% Triton X-100, 0.5% Tween 20, 0.25 mM EDTA and 1 mg/mL proteinase K) on the wall of a 0.2 mL PCR tube. The tube was briefly centrifuged to bring the worm to the bottom of the tube and then incubated at 65°C for 10 min, followed by 1 min at 85°C to inactivate proteinase K, in a thermocycler (Eppendorf) and then immediately cooled on ice. Before cDNA synthesis, genomic DNA digestion was performed using Maxima H Minus cDNA synthesis kit (Thermo Fisher, Waltham, MA, USA) as described in the manufacturer’s manual. Then, 10 μl of cDNA synthesis mix were added to the worm lysate. The final mix contains 1X supplier-provided buffer, 0.5 mM dNTP mix, 5 μM random hexamer primers and 1X Maxima H minus reverse transcriptase Mix. The tube was gently mixed, briefly centrifuged, and incubated at 25°C for 10 min, followed by 55°C for 30 min and a final 85°C for 5 min. The cDNA was diluted at 2.5X with H2O and used immediately for qPCR analysis.

#### Quantification of mRNA expression by RT-PCR

Real-time quantitative polymerase chain reaction (RT-qPCR) was performed using a ViiA7 Sequence Detection System instrument and software (Life Technologies, Applied Biosystems, Courtaboeuf, France). Gene-specific primers for SYBR Green assays were designed according to the genome sequence of *C*. *elegans* (www.wormbase.org), using Primer Express software version 2.0 (Applied Biosystems) and synthesized by Invitrogen (Cergy Pontoise, France). All primers were entered into the NCBI Blast program to ensure specificity. Results were expressed using the comparative Ct method as described in User Bulletin 2 (Applied Biosystem). Briefly, the ΔC_t_ values were calculated in every sample for each gene of interest as following: C_t gene of interest_—C_t reporter gene_, with cell division cycle protein 42 (CDC42) as the reference gene. The relative expression of the target genes was calculated using the comparative 2^-ΔΔCt^ method [80]. A dissociation curve allowed us to verify the specificity of the amplification.

### Generation of transgenic *C*. *elegans*

#### Cloning the promoter *Cel-nhr-8p* and the complete coding cDNA sequences of *nhr-8* from *C*. *elegans* and *H*. *contortus*

Nested PCR amplifications were performed with the proofreading Phusion High-Fidelity DNA Polymerase (New England Biolabs, Ipswich, MA, USA) following the manufacturer’s recommendation. All primers used are listed in S1 Table. *Cel-nhr-*8p and *Cel-nhr-*8 full length were amplified using gDNA from Bristol N2 *C*. *elegans* adult worms as template, while *Hco-nhr-8* full length was amplified using cDNA prepared from *H*. *contortus* L2 larvae (Weybridge isolate), respectively. Primers 5’-ctgcagACCAAGTGCAGGATTACGATGA-3’ and 5’-cccgggGGAATGACGAAATTTTTGTTT-3’ were used to amplify a 3101 bp promoter region as previously described [31]. This promoter region was cloned into pPD95.75 (Addgene) between *PstI* and *SmaI* sites. Complete coding sequences corresponding to *Cel-nhr-8* and *Hco-nhr-8* genes were obtained using 5’-cccgggATGCCTTCGTCTTCTCCATC-3’ and 5’-cccgggCATGGTTAATAAATGGTTATTCA-3’ for *C*. *elegans* and 5’-cccgggATGACACAACTCTCACCAGAG-3’ and 5’-cccgggTCAAATCATATCGAACAACTCTT-3’ for *H*. *contortus*. These amplicons were inserted into plasmid pPD95.75 containing *Cel-nhr-8p* using restriction site *SmaI*. All constructs were purified using the GenElute PCR Clean-Up Kit (Sigma-Aldrich, Saint-Quentin-Fallavier, France) and sequenced at the GeT-PlaGe core facilities of Genotoul (Toulouse, France).

#### Cloning the promoter *Cel-eft-3p* and the complete coding cDNA sequences of *pgp-6* from *C*. *elegans*

The *Cel-eft-3* promoter region was cloned into pUC57 using Gibson assembly. *Cel-pgp-6* full length was amplified using gDNA from Bristol N2 *C*. *elegans* adult worms as template. This amplicon was inserted into pUC57 containing Cel-eft-3p using Gibson assembly to generate *Cel-eft-3p*::*Cel-pgp-6*::*tbb-2u*. All constructs were purified using the GenElute PCR Clean-Up Kit (Sigma-Aldrich, Saint-Quentin-Fallavier, France) and sequenced at Nemametrix (USA, OR).

#### Transformation of *C*. *elegans*

Young adult hermaphrodite *nhr-8(ok186) C*. *elegans* were transformed by microinjection of plasmids into the gonads as described previously [81,82]. Different mixes containing modified pPD95.75 plasmids containing *Cel-nhr-8p*::*Cel-nhr-8* (40 ng/μl), *Cel-nhr-8p*::*Hco-nhr-8* (40 ng/μl), *Cel-eft-3p*::*Cel-pgp-6*::*tbb-2u* (15 ng/μl) or the untransformed pPD95.75 and pUC57 (15 ng/μl) were injected with the plasmid pCFJ104_*myo3p*::*mCherry* (Addgene, 10 ng/μl in injection mix) as a transformation marker. The total concentration of DNA in the injection mix was brought to 100ng/ul using salmon testis DNA. Successful transformation was determined by identification of the selection marker under a SMZ800 fluorescent stereomicroscope (Nikon, France). At least three independent lines carrying extra chromosomal arrays were obtained, for each construct. Only worms expressing the mCherry were selected for pharmacological analysis of pharyngeal pumping. Transcript levels of *Cel-nhr-8* and *Hco-nhr-8* were evaluated in each independent lines by single worm qRT-PCR (S7 Fig).

### Electropharyngeograms (EPGs) recording and pumping activity analysis

Synchronized worms (wild-type Bristol N2; AE501 *nhr-8(ok186)*; *Cel-nhr-8* in AE501*; Hco-nhr-*8 in AE501; *Cel-pgp-6* in AE501) were cultivated at 21°C to the first day of adulthood on plates containing standard nematode growth medium (NGM) seeded with *E*. *coli* OP50. A stock solution of 100 μM IVM was prepared in DMSO. 1 μL of this stock was added to 1 mL of M9 buffer containing 10 mM of the neuromodulator serotonin (5-hydroxytryptamine; 5HT) to stimulate pumping [83]. The final concentrations in the IVM solution were 0.1 μM IVM, 0.1% DMSO and 10 mM 5HT. The solvent control solution contained 0.1% DMSO and 10 mM 5HT. The 10 mM 5HT solution in M9 was used within 4 h. Day 1 adults were collected from a plate using M9 buffer, washed twice with M9 by centrifugation (2 min, 6,000 RPM) and incubated for 20 min with M9 containing 10 mM 5HT to activate pharyngeal pumping. Then, IVM, or DMSO for the control group, was added. After 20 min incubation, pump frequency was measured from EPG recordings using the NemaMetrix ScreenChip 40 system [83]. Each EPG recording was 2 to 4 min in duration and the experiment was replicated at least in triplicate on different days. EPG recordings were started ~5 min after the onset of IVM exposure and continued for an additional 60 min.

### Assay of ABC transporter-mediated xenobiotic transport in *C*. *elegans*

To evaluate some active transport of xenobiotics mediated by ABC transporters in *C*. *elegans*, we used a PGP functional assay that has been developed in mammalian cells [84] and adapted in nematodes [85]. Accumulation of the fluorescent PGP substrate rhodamine 123 (rho123) was investigated in adult wild-type Bristol N2 and *nhr-8(ok186) C*. *elegans*. Briefly, synchronized worms were collected in M9 and incubated in with rho123 at 10 μM for 48 h at 21°C under gentle agitation. After a wash with M9 and a short recovery period on NGM plates, worms were picked, paralyzed using levamisole (2.5 mM) and observed by using a Nikon Eclipse 50i microscope equipped with a Luca S camera and analyzed using Nikon ACT-1 software. Image analysis was adapted from a method previously described [86]. Briefly, images were taken for each individual animal. For each strain, 30 to 40 worms were imaged. On each image, an area of interest was drawn and the number of pixels corresponding to the area was determined in IMAGEJ (US National Institutes of Health, Bethesda, MD, USA; http://imagej.nih.gov/ij/). Then, the area occupied by rho123 fluorescence for each 2D image was determined. The ratio between the number of pixels corresponding to the area occupied by rho123 and the number of pixel corresponding to the total area of the worm was determined and used to compare rho123 accumulation in the two strains. Results were reported as quantity of rho123 fluorescence for each individual animal and shown as mean ± SEM.

### Statistical analysis

All experiments were conducted independently at least in triplicate and results are expressed as mean ± standard deviation (S.D.) unless otherwise stated. Statistical analysis was performed using one-way analysis of variance (ANOVA) with a Tukey post-test to compare the effect of IVM over control or *nhr-8(ok186)* over wild-type, while individual comparisons between pairs of data were performed using the Mann-Whitney test (GraphPad Instat, San Diego, CA, USA). Statistical significance was accepted as p<0.05.

## Supporting information

S1 Fig Dose response curves to IVM in a larval development assay of three *C*. *elegans nhr-8* loss-of-function strains (*nhr-8(ok186)*, *nhr-8(hd117)* and *nhr-8(tm1800))* in comparison to the wild-type Bristol N2. Values represent the percentage of L1 reaching the young adult stage after 55 hours of incubation at 21°C within the presence of increasing doses of IVM. Data are mean ± SEM from four independent experiments.
(TIF)

S2 Fig Quantification of *nhr-8* transcript levels in *nhr-8* RNAi *C*. *elegans*. Real-time RT-PCR analysis was applied after RNAi treatment with control RNAi or specific *nhr-8* RNAi. *Nhr-8* mRNA level value in wild-type strain in control animals fed bacteria containing empty vector (CTR RNAi) is set to 1. *Nhr-8* mRNA level in IVM-selected (IVR10) and MOX-selected is expressed as fold change relative to CTR RNAi. Data were normalized against *cdc-42* as an internal control and are mean ± S.D. from four independent RNA preparations for each strain.
(TIF)

S3 Fig Expression levels of genes associated with IVM resistance in *nhr-8(ok186) C*. *elegans* relative to wild-type worms. Changes in levels of mRNAs encoding genes known to be associated with IVM resistance, normalized with respect to *cdc-42* mRNA levels, were determined by real-time qPCR. Gene expression levels are expressed as -fold change relative to wild-type Bristol N2 and are reported as the mean ± S.D. of three to four independent experiments. No significant difference *vs*. wild-type Bristol N2.
(TIF)

S4 Fig Dye filling of amphid neurons in Bristol N2 and *nhr-8(ok186)* strains. Young adults *C*. *elegans* from wild-type Bristol N2 and AE501 *nhr-8(ok186)* strains were examined by fluorescent microscopy to visualize the dye-filling of the amphidial dendrites after staining with the fluorescent dye DiIC_12_(3). Arrows indicate the amphidial dendrites.
(TIF)

S5 Fig Percentage of *C*. *elegans* with dye-filling defective amphid neurons phenotype. Dye-filling defect of amphid neurons in the wild-type Bristol N2 and IVM-resistant strains was analysed after gene-specific silencing of *nhr-8* (grey bar) and compared with control RNAi (see Material & methods section for the RNAi technique). Young adults worms were examined by fluorescence microscopy to visualize the dye filling of the amphidial dendrites after staining with the fluorescent dye DiIC12(3) (DiI) and scored for the dye filling (Dyf) defective phenotype (absence of red staining of the amphid neuron). Data are mean ± S.D.
(TIF)

S6 Fig Sequence comparison of NHR-8 from *C*. *elegans* (Accession No. NP_741445) and *H*. *contortus* (Accession No. Q9XYB7_Hco). The alignments were performed using ClustalO and LALIGN and refined manually. **(A&B) Protein sequence alignments of (A) DNA binding domains (DBD) and (B) Ligand binding domain (LBD) of Hco-NHR-8 and Cel-NHR-8**. The two zinc fingers (CI and CII) and the conserved motifs are shown. Conserved cysteines that comprise Zn coordination sites of the DBD are starred (black boxes). CI: the first zinc finger with a conserved motif sequence of Cys-X2-Cys-X13-Cys-X2-Cys; CII: the second zinc finger with a conserved motif sequence of Cys-X5-Cys-X9-Cys-X2-Cys. Cys: cysteine residue, X followed with a number indicates the number of amino acids between the Cys. The dotted boxes indicate the amino acid sequences of P and D boxes. In the LBD, positions of the 12 α helices (H1-H12) are shown. Consensus symbols: * (asterisk, dark grey): positions with fully conserved residue; : (colon, grey): conservation between amino acids having closely similar characteristics (scoring > 0.5 in the Gonnet PAM 250 matrix); . (period): conservation between amino acids with weak similarity (scoring = < 0.5 in the Gonnet PAM 250 matrix). At the end of each row the amino acid numbers are given for the particular protein.
(TIF)

S7 Fig Quantitative RT-PCR of *Cel-nhr-8* and *Hco-nhr-8* transcript levels in wild-type Bristol N2 and transgenics *C*. *elegans* (*Cel-nhr-8* in AE501 and *Hco-nhr-*8 in AE501). mRNA expression level of *nhr-8*, normalized with respect to *cdc-42* mRNA levels, was evaluated on individual worms by qRT-PCR. *Cel-nhr-8* mRNA level was evaluated in wild-type and the transgenic line “*Cel-nhr-8* in AE501”, while *Hco-nhr-8* mRNA level was evaluated in the transgenic line “*Hco-nhr-8* in AE 501”. Data are expressed as fold change relative to the expression level on *Cel-nhr-8* in wild-type worms, which is set to 1, and are the mean of 8 to 24 worms per strain. * p<0.05; vs. wild-type Bristol N2.
(TIF)

S8 Fig Expression of *Hco-nhr-8* in the free-living stages of *Haemonchus contortus*. Level of transcription of *Hco-nhr-8* throughout the free-living stages of *H*. *contortus* was investigated by qRT-PCR; Eggs: embryonated egg; L2: second stage larvae; L3: third stage larvae. *Hco-nhr-8* mRNA levels were normalized with respect to the *H*. *contortus gapdh* mRNA levels, and are expressed as -fold change relative to the expression level in eggs.
(TIF)

S1 Table Sequences of primers used in the study. (DOCX)

S2 Table Percentage of *nhr-8* transcript levels remaining after *nhr-8* silencing through RNAi. (DOCX)
