# Supplementary figures and images for: The transcription factor NHR-8: A new target to increase ivermectin efficacy in nematodes
Source: PLoS Pathog. 2019 Feb 13;15(2):e1007598. doi: 10.1371/journal.ppat.1007598 (PMC6391013; doi:10.1371/journal.ppat.1007598)

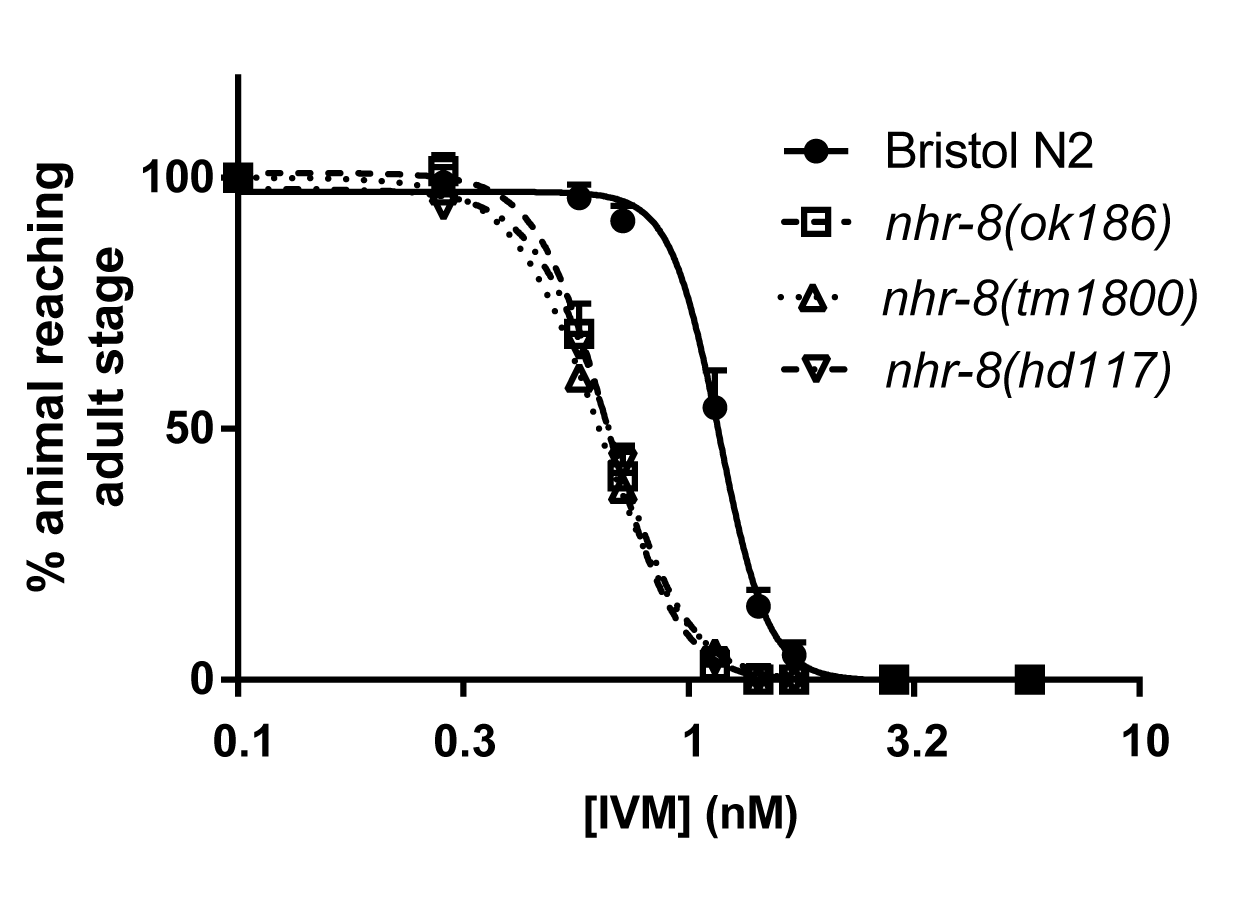

Supplement: S1 Fig — Values represent the percentage of L1 reaching the young adult stage after 55 hours of incubation at 21°C within the presence of increasing doses of IVM. Data are mean ± SEM from four independent experiments. (TIF) [file ppat.1007598.s001.tif]

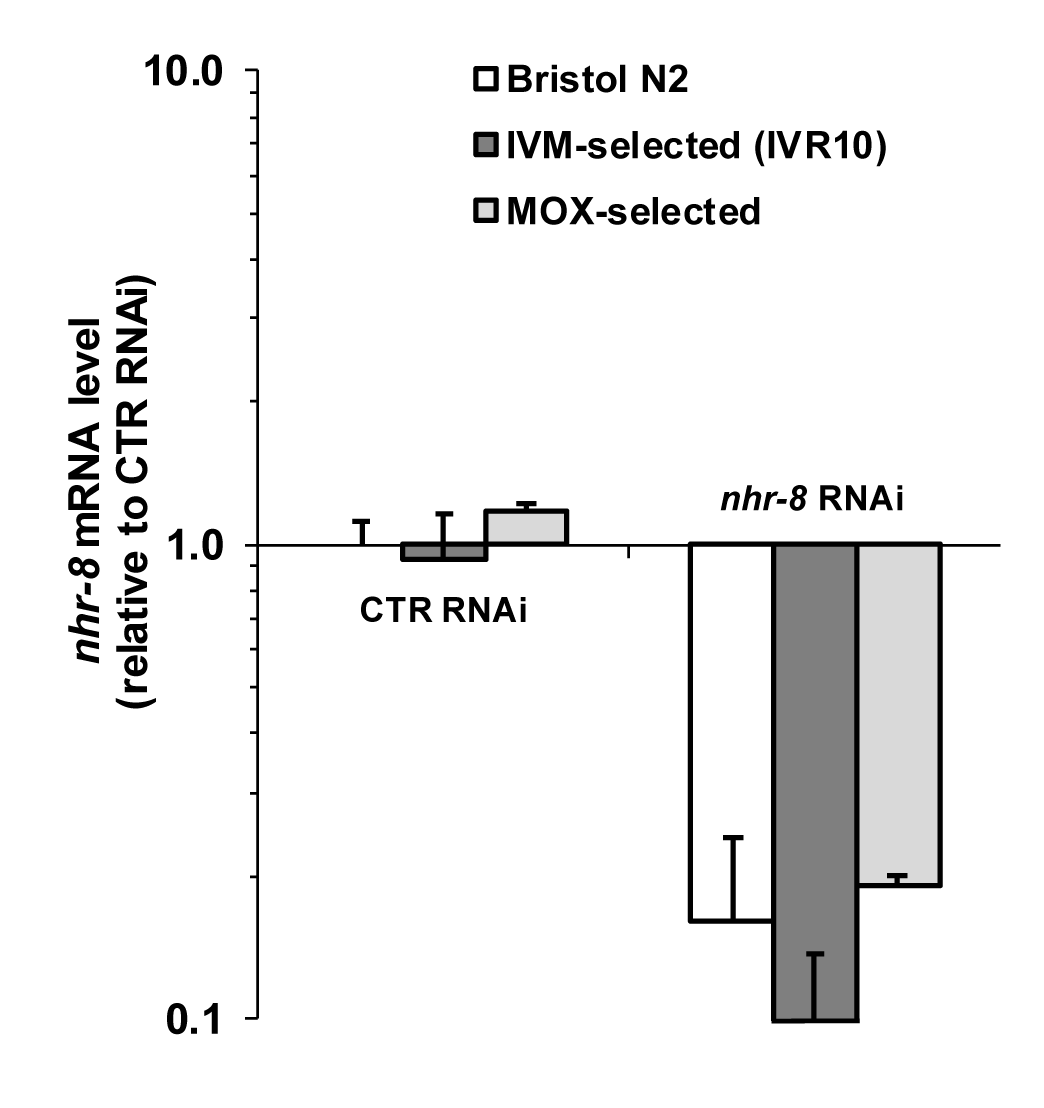

Supplement: S2 Fig — Real-time RT-PCR analysis was applied after RNAi treatment with control RNAi or specific nhr-8 RNAi. Nhr-8 mRNA level value in wild-type strain in control animals fed bacteria containing empty vector (CTR RNAi) is set to 1. Nhr-8 mRNA level in IVM-selected (IVR10) and MOX-selected is expressed as fold change relative to CTR RNAi. Data were normalized against cdc-42 as an internal control and are mean ± S.D. from four independent RNA preparations for each strain. (TIF) [file ppat.1007598.s002.tif]

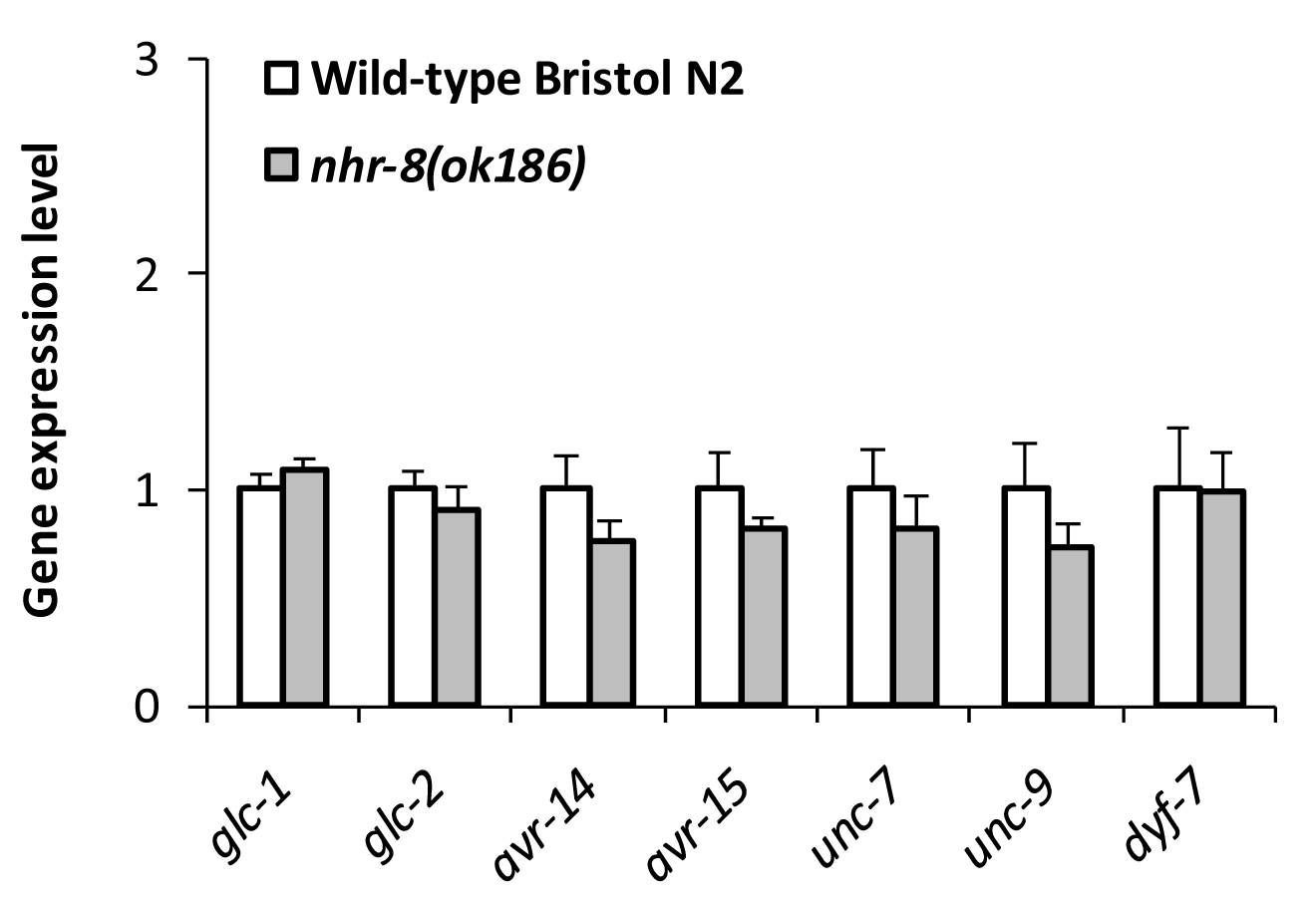

Supplement: S3 Fig — Changes in levels of mRNAs encoding genes known to be associated with IVM resistance, normalized with respect to cdc-42 mRNA levels, were determined by real-time qPCR. Gene expression levels are expressed as -fold change relative to wild-type Bristol N2 and are reported as the mean ± S.D. of three to four independent experiments. No significant difference vs. wild-type Bristol N2. (TIF) [file ppat.1007598.s003.tif]

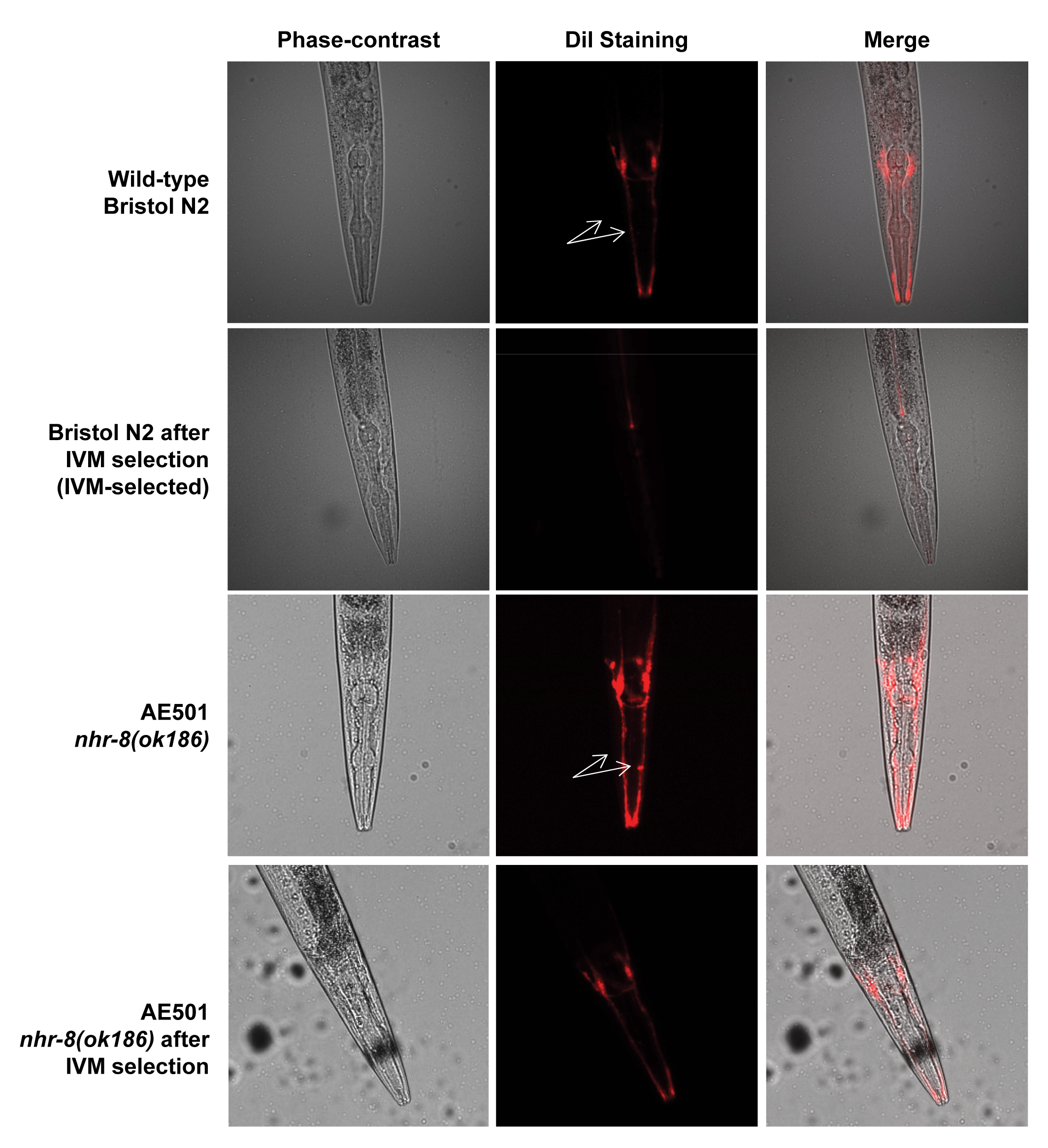

Supplement: S4 Fig — Young adults C. elegans from wild-type Bristol N2 and AE501 nhr-8(ok186) strains were examined by fluorescent microscopy to visualize the dye-filling of the amphidial dendrites after staining with the fluorescent dye DiIC12(3). Arrows indicate the amphidial dendrites. (TIF) [file ppat.1007598.s004.tif]

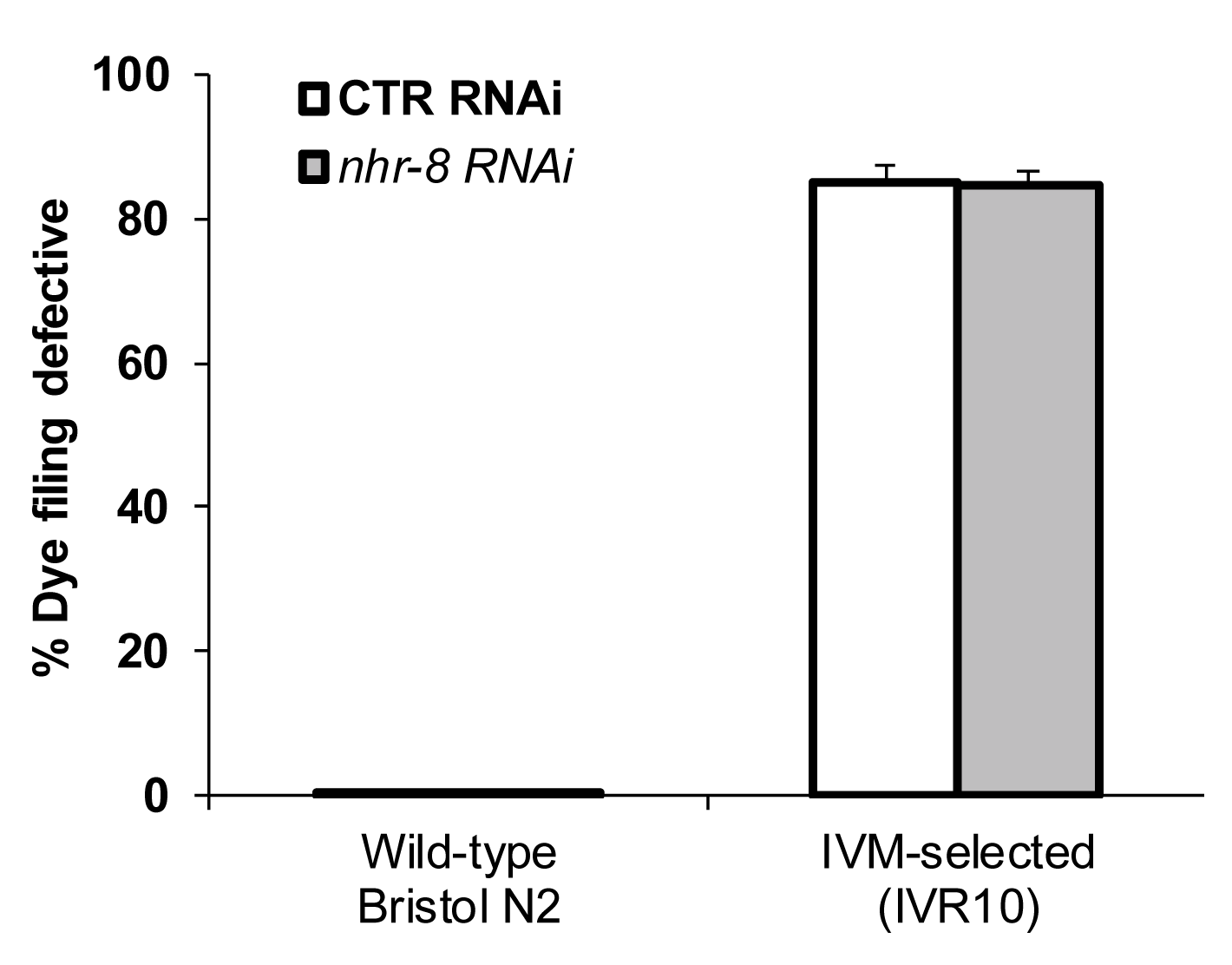

Supplement: S5 Fig — Dye-filling defect of amphid neurons in the wild-type Bristol N2 and IVM-resistant strains was analysed after gene-specific silencing of nhr-8 (grey bar) and compared with control RNAi (see Material & methods section for the RNAi technique). Young adults worms were examined by fluorescence microscopy to visualize the dye filling of the amphidial dendrites after staining with the fluorescent dye DiIC12(3) (DiI) and scored for the dye filling (Dyf) defective phenotype (absence of red staining of the amphid neuron). Data are mean ± S.D. (TIF) [file ppat.1007598.s005.tif]

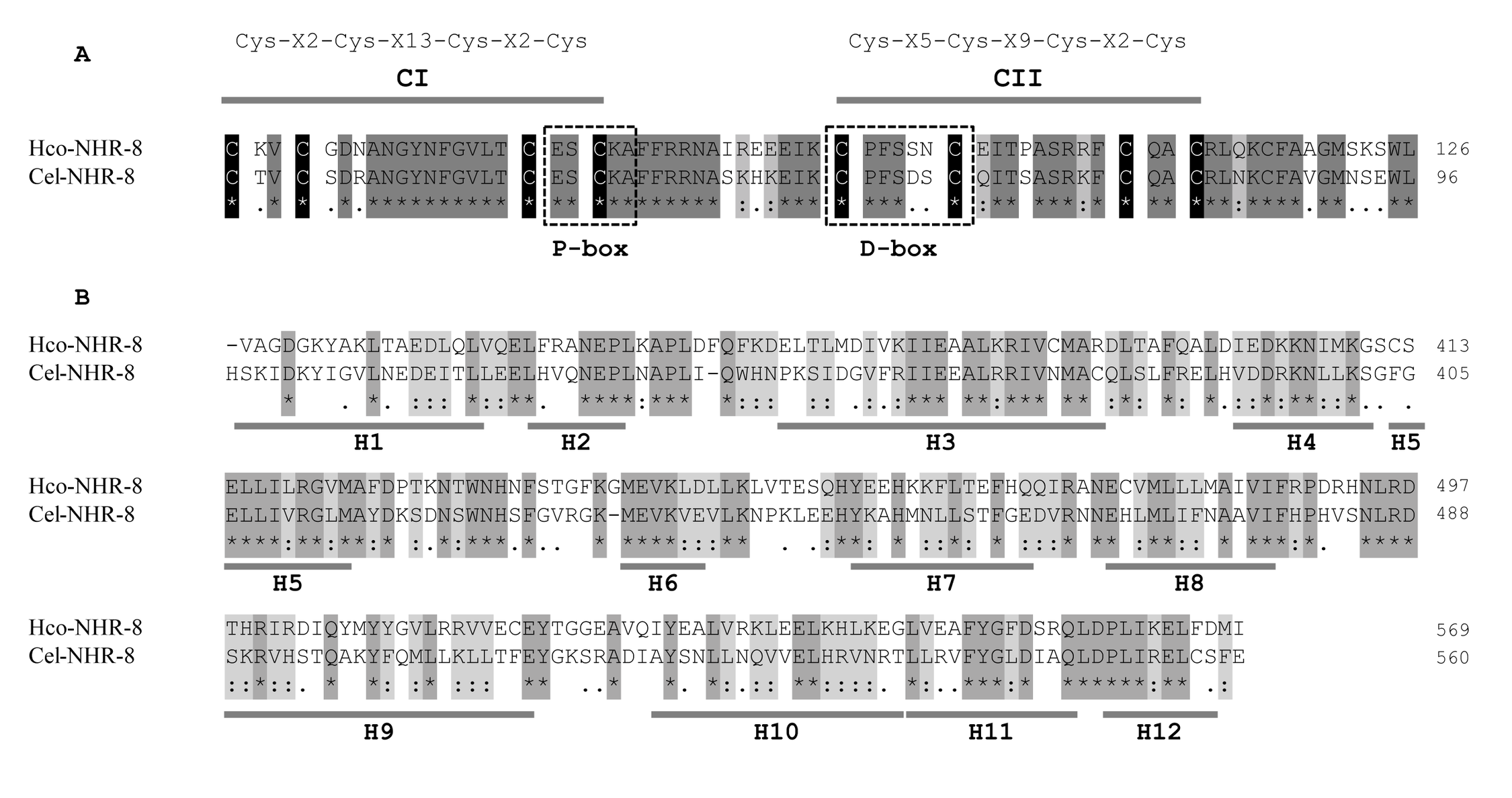

Supplement: S6 Fig — The alignments were performed using ClustalO and LALIGN and refined manually. (A&B) Protein sequence alignments of (A) DNA binding domains (DBD) and (B) Ligand binding domain (LBD) of Hco-NHR-8 and Cel-NHR-8. The two zinc fingers (CI and CII) and the conserved motifs are shown. Conserved cysteines that comprise Zn coordination sites of the DBD are starred (black boxes). CI: the first zinc finger with a conserved motif sequence of Cys-X2-Cys-X13-Cys-X2-Cys; CII: the second zinc finger with a conserved motif sequence of Cys-X5-Cys-X9-Cys-X2-Cys. Cys: cysteine residue, X followed with a number indicates the number of amino acids between the Cys. The dotted boxes indicate the amino acid sequences of P and D boxes. In the LBD, positions of the 12 α helices (H1-H12) are shown. Consensus symbols: * (asterisk, dark grey): positions with fully conserved residue; : (colon, grey): conservation between amino acids having closely similar characteristics (scoring > 0.5 in the Gonnet PAM 250 matrix); . (period): conservation between amino acids with weak similarity (scoring = < 0.5 in the Gonnet PAM 250 matrix). At the end of each row the amino acid numbers are given for the particular protein. (TIF) [file ppat.1007598.s006.tif]

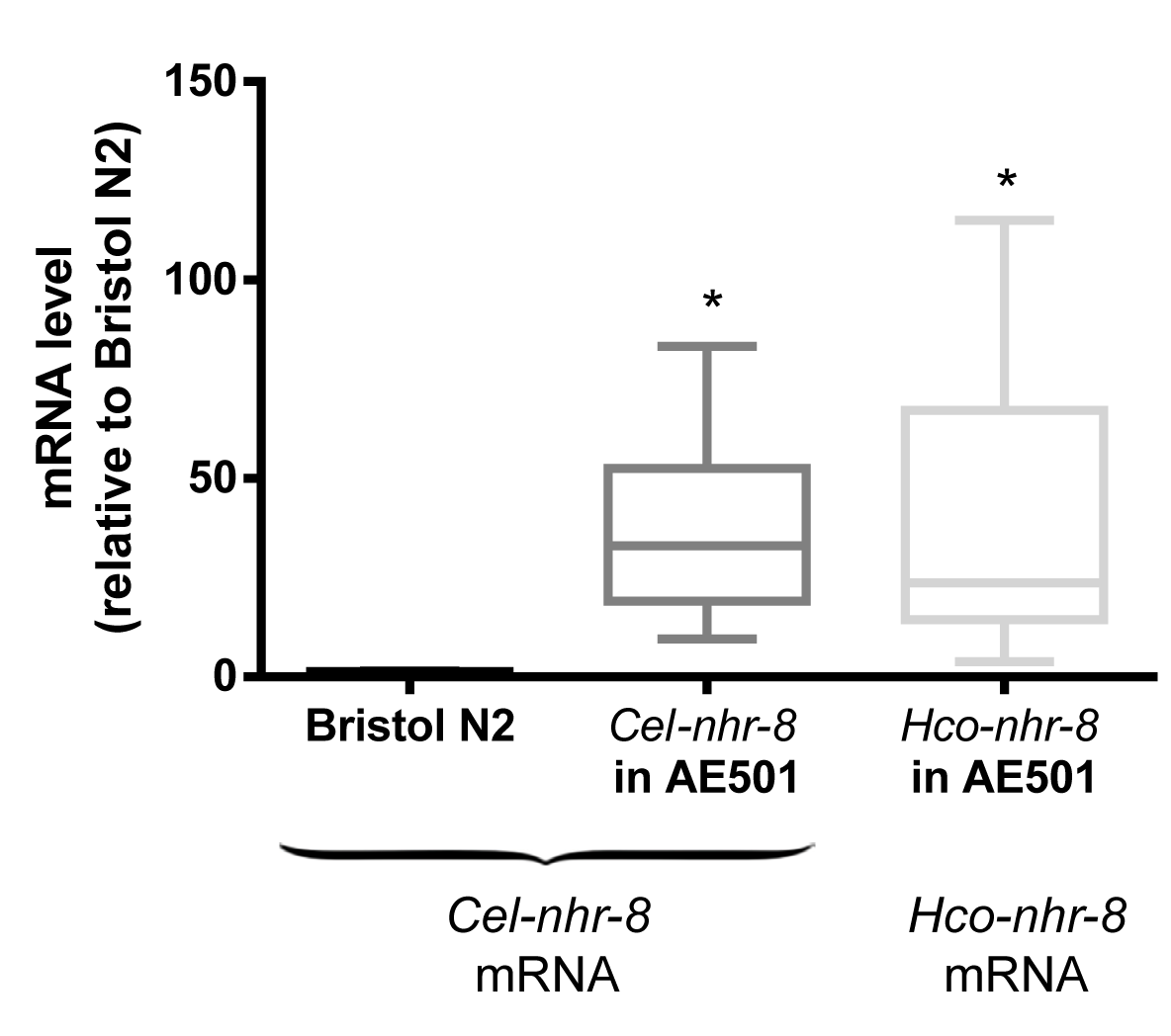

Supplement: S7 Fig — mRNA expression level of nhr-8, normalized with respect to cdc-42 mRNA levels, was evaluated on individual worms by qRT-PCR. Cel-nhr-8 mRNA level was evaluated in wild-type and the transgenic line “Cel-nhr-8 in AE501”, while Hco-nhr-8 mRNA level was evaluated in the transgenic line “Hco-nhr-8 in AE 501”. Data are expressed as fold change relative to the expression level on Cel-nhr-8 in wild-type worms, which is set to 1, and are the mean of 8 to 24 worms per strain. * p<0.05; vs. wild-type Bristol N2. (TIF) [file ppat.1007598.s007.tif]

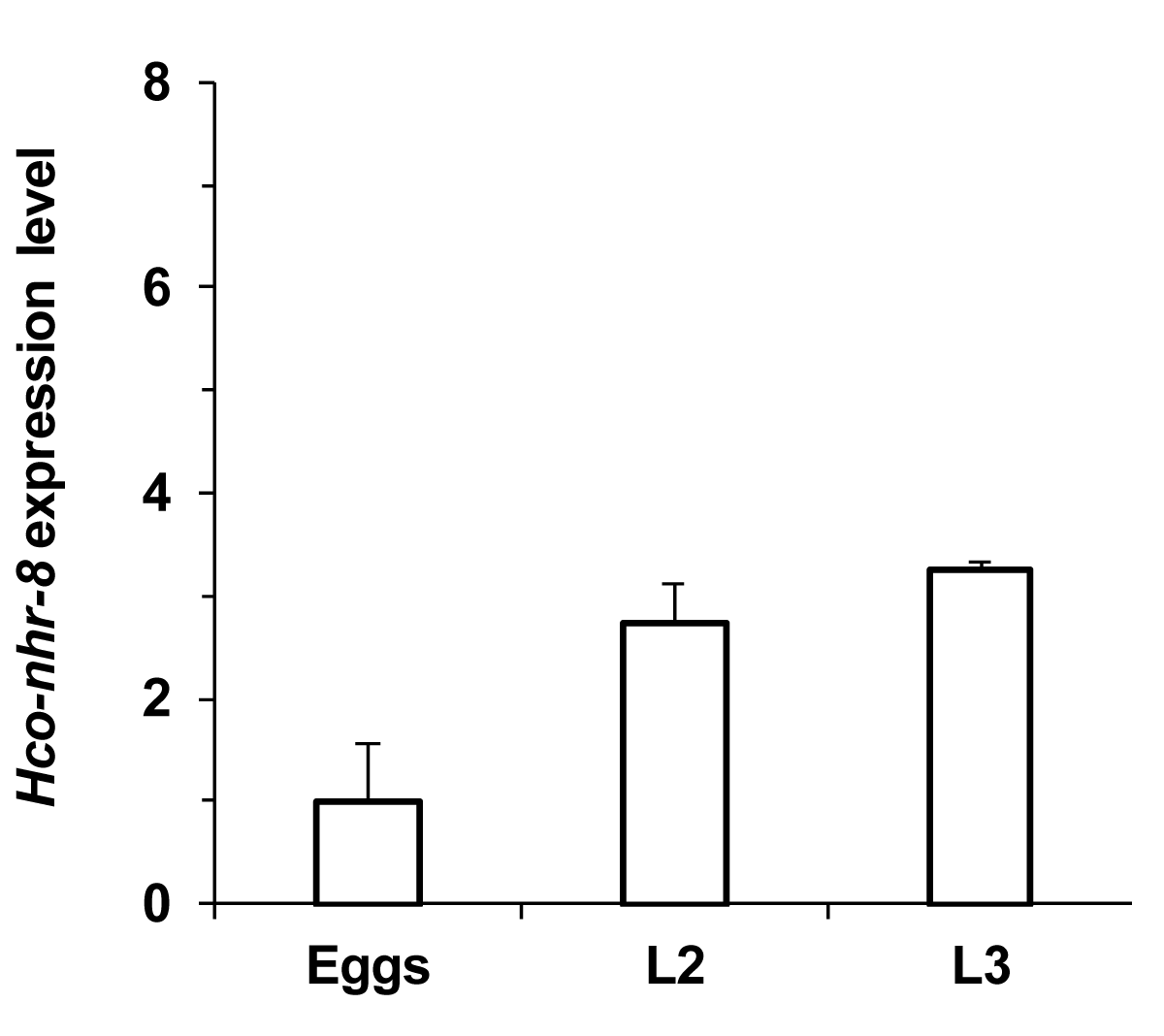

Supplement: S8 Fig — Level of transcription of Hco-nhr-8 throughout the free-living stages of H. contortus was investigated by qRT-PCR; Eggs: embryonated egg; L2: second stage larvae; L3: third stage larvae. Hco-nhr-8 mRNA levels were normalized with respect to the H. contortus gapdh mRNA levels, and are expressed as -fold change relative to the expression level in eggs. (TIF) [file ppat.1007598.s008.tif]
