# Supplementary material for: The transcription factor NHR-8: A new target to increase ivermectin efficacy in nematodes
Source: PLoS Pathog. 2019 Feb 13;15(2):e1007598. doi: 10.1371/journal.ppat.1007598 (PMC6391013; doi:10.1371/journal.ppat.1007598)
Supplement: S1 Table — (DOCX) [file ppat.1007598.s009.docx]

**S1 Table. Sequences of primers used in the study.**

| Primer name | 5'-3' sequence |
| --- | --- |
| *pCel-nhr8-*WF0 | TCTTCTCCTGGAATTGTCATCAA |
| *pCel-nhr-8-*WR0 | GCAGACCTTCGAGACTCGT |
| *pCel-nhr8-*FPstI | tttttctgcagACCAAGTGCAGGATTACGATGA |
| *pCel-nhr-8-*RSmaI | gggggcccgggGGAATGACGAAATTTTTGTTT |
| *Cel-nhr-8-*WR0 | CTCCATCTTTCTGCCCTTGACT |
| *Cel-nhr-8-*WF0 | TCCACTTGTGGTGTGACTTATC |
| *Cel-nhr-8-*FSmaI | tttttcccgggATGCCTTCGTCTTCTCCATC |
| *Cel-nhr-8-*RSmaI | tttttcccgggCATGGTTAATAAATGGTTATTCA |
| SL1 | GGTTTAATTACCCAAGTTTGAG |
| *Hco-nhr-8-*WR0 | ATGAATCATATTAAGCAAGGCT |
| *Hco-nhr-8*-FSmaI | tttttcccgggATGACACAACTCTCACCAGAG |
| *Hco-nhr-8-*RSmaI | gggggcccgggTCAAATCATATCGAACAACTCTT |
