# Supplementary material for: The transcription factor NHR-8: A new target to increase ivermectin efficacy in nematodes
Source: PLoS Pathog. 2019 Feb 13;15(2):e1007598. doi: 10.1371/journal.ppat.1007598 (PMC6391013; doi:10.1371/journal.ppat.1007598)
Supplement: S2 Table — (DOCX) [file ppat.1007598.s010.docx]

**S2 Table. Percentage of *nhr-8* transcript levels remaining after *nhr-8* silencing through RNAi.** RNAs were extracted from worms fed on HT115 bacteria transformed whether with L4440 vector that produces double-stranded RNA against *Cel-nhr-8*, or with the empty vector as control. mRNA levels were determined by real-time qPCR.

| Strains | *nhr-8* mRNA level  (% relative to control RNAi) |
| --- | --- |
| **Wild-type Bristol N2** | 16.0 ± 8.0 |
| **IVM-selected (IVR10)** | 9.7 ± 4.6 |
| **MOX-selected** | 16.2 ± 1.9 |
